# Classical MHC expression by DP thymocytes impairs the selection of non-classical MHC restricted innate-like T cells

**DOI:** 10.1038/s41467-021-22589-z

**Published:** 2021-04-16

**Authors:** Hristo Georgiev, Changwei Peng, Matthew A. Huggins, Stephen C. Jameson, Kristin A. Hogquist

**Affiliations:** 1 Center for Immunology and Department of Laboratory Medicine and Pathology, University of Minnesota, Minneapolis, MN 55455 USA; 2 Present Address: Institute of Immunology, Hannover Medical School, Hannover, D-30625 Germany

**Keywords:** MHC, Innate immunity, NKT cells, Thymus

## Abstract

Conventional T cells are selected by peptide-MHC expressed by cortical epithelial cells in the thymus, and not by cortical thymocytes themselves that do not express MHC I or MHC II. Instead, cortical thymocytes express non-peptide presenting MHC molecules like CD1d and MR1, and promote the selection of PLZF^+^ iNKT and MAIT cells, respectively. Here, we report an inducible class-I transactivator mouse that enables the expression of peptide presenting MHC I molecules in different cell types. We show that MHC I expression in DP thymocytes leads to expansion of peptide specific PLZF^+^ innate-like (PIL) T cells. Akin to iNKT cells, PIL T cells differentiate into three functional effector subsets in the thymus, and are dependent on SAP signaling. We demonstrate that PIL and NKT cells compete for a narrow niche, suggesting that the absence of peptide-MHC on DP thymocytes facilitates selection of non-peptide specific lymphocytes.

## Abstract (web summary)

Conventional T cell subsets are selected in the thymus by peptide bearing MHC expressed by cortical epithelial cells, in contrast cortical thymocytes express non-peptide bearing MHC molecules including CD1d and MR1 and select iNKT and MAIT cell populations respectively. Here, the authors generate a novel inducible MHC class-I trasnactivator murine system and suggest the absence of peptide-MHC on thymocytes is involved in the selection of non-peptide specific lymphocytes.

## Introduction

The study of innate-like T cells, namely invariant natural killer T (iNKT) cells, mucosal-associated invariant T (MAIT) cells, and γδ T cells, is rapidly expanding and gaining interest. Defined by their phenotype, innate-like T cells bear functional T-cell receptor (TCR) rearrangements, yet acquire a memory phenotype during development, and quickly respond to stimulation without undergoing extensive clonal expansion^1,2^. They are generally thought to recognize conserved antigens^3^. For these reasons, they are considered as a bridge between adaptive and innate immunity, and are seen as an appealing target for immune therapies. As a prominent member of this group, iNKT cells were initially described as cells with such unconventional phenotype^4–7^. Consequently, it was shown that iNKT cells share the same developmental path with conventional αβ T cells until the double-positive (DP) thymocyte stage^8^. Yet, they follow a different process of thymic selection. Conventional αβ T cells undergo positive and negative selection by thymic epithelial cells (TECs) presenting peptide antigens by classical class I and II major histocompatibility complex (MHC) molecules. In contrast iNKT cells are positively selected on hematopoietic cells (HCs), in particular, another DP thymocyte presenting lipid antigens via the non-classical MHC-like molecule CD1d^3,9–11^. In addition, positive selection of iNKT cells requires a strong TCR signaling, a process known as “agonist selection,” and a secondary co-stimulatory signal provided by homotypic interactions between signaling lymphocyte activation molecule family (SLAMF) receptors^12,13^. As a result, selected cells upregulate the key transcription factor PLZF, which governs the commitment to the iNKT cell lineage^14,15^. Subsequently, more innate-like T-cell subsets were described, including PLZF^+^ γδ T cells and MAIT cells. Similar to iNKT cells, both cell subsets express PLZF and the latter were shown to be positively selected by DP thymocytes^16,17^. In addition, it was reported that non-classical MHC Ib-restricted cells can be selected by TECs or by HCs. However, they develop an innate-like phenotype only when they are selected on HCs but not on TECs^18^. Thus, it appears that a common feature of innate-like T cells is being positively selected by thymocytes. Thus, DP–DP interactions are proven to be pivotal for induction of PLZF expression and initiation of an innate-like program in T cells. Yet, the underlining mechanisms governing selection and development of these cells are still elusive.

Strikingly, murine DP thymocytes do not express classical MHC I or MHC II molecules^19,20^. It is possible that MHC downregulation at this stage could be a mechanism preventing high levels of diversion to a PLZF-expressing innate-like T-cell lineage, which could compromise conventional CD4 and CD8 T-cell repertoire development. Alternatively, it might facilitate greater development and selection of non-classical MHC I-restricted T cells by reducing competition. Two independent groups generated transgenic mice where MHC II expression was forced at the DP thymocyte stage^21,22^. Both the mouse lines were generated by expressing the human class II MHC transactivator (CIITA) under the control of CD4 or proximal Lck promotor regions. Consequently, in both transgenic mice, MHC II was expressed on DP thymocytes. This lead to an expansion of thymocyte-selected PLZF^+^ T cells with an innate-like phenotype similar to iNKT cells^23,24^.

Yet, innate-like T cells, such as iNKT and MAIT cells, are naturally selected on thymocytes expressing non-classical members of the MHC I family. Thus, we wanted to investigate the effects of forced expression of classical MHC I. Recently, the Class I transactivator (CITA, also called Nlrc5) was reported to be a positive transcriptional regulator of MHC I expression^25–27^. In addition, Nlrc5 drives transcription of components necessary for MHC I processing and peptide loading such as β2m, Tap1, and Lmp2^25,28^. Here we show that Nlrc5 is not expressed in DP thymocytes, despite their high expression of CD1d. We describe an inducible transgenic mouse where murine Nlrc5 can be specifically expressed in different cell types. Remarkably, forced Nlrc5 expression in DP thymocytes led to a dramatic upregulation of peptide-presenting MHC I molecules on the cell surface, therefore making them potentially capable of selecting and steering peptide-specific MHC I-restricted T cells into an innate-like T-cell developmental pathway. Indeed, similar to MHC II, MHC I expression on DP thymocytes provoked expansion of PLZF^+^ innate-like (PIL) T cells. Moreover, both MHC I- and II-restricted PIL T cells displayed a phenotype resembling iNKT cells in that they were found to exist in three functional subsets based on the expression pattern of characteristic transcription factors, cytokines, and surface markers, and their development required the SLAM-associated adaptor protein (SAP). By analyzing bone marrow (BM) chimeras, PIL T-cell selection was attributed to MHC I expression on neighboring DP thymocytes and not to a cell-intrinsic effect. Although conventional CD4 and CD8 T-cell development was not compromised in these mice, the development of iNKT cells was reduced, indicating that PIL T cells and iNKT cells compete for a limited thymic niche. These results suggest that the absence of peptide-presenting MHC class I molecules on DP thymocytes is a consequence of not expressing Nlrc5 and functions to facilitate the development of non-peptide-specific lymphocyte subsets such as iNKT cells.

## Results

### Generation of an Nlrc5-stop^flox^ transgenic mouse as a tool for conditional expression of MHC class I molecules

**Fig. 1:**
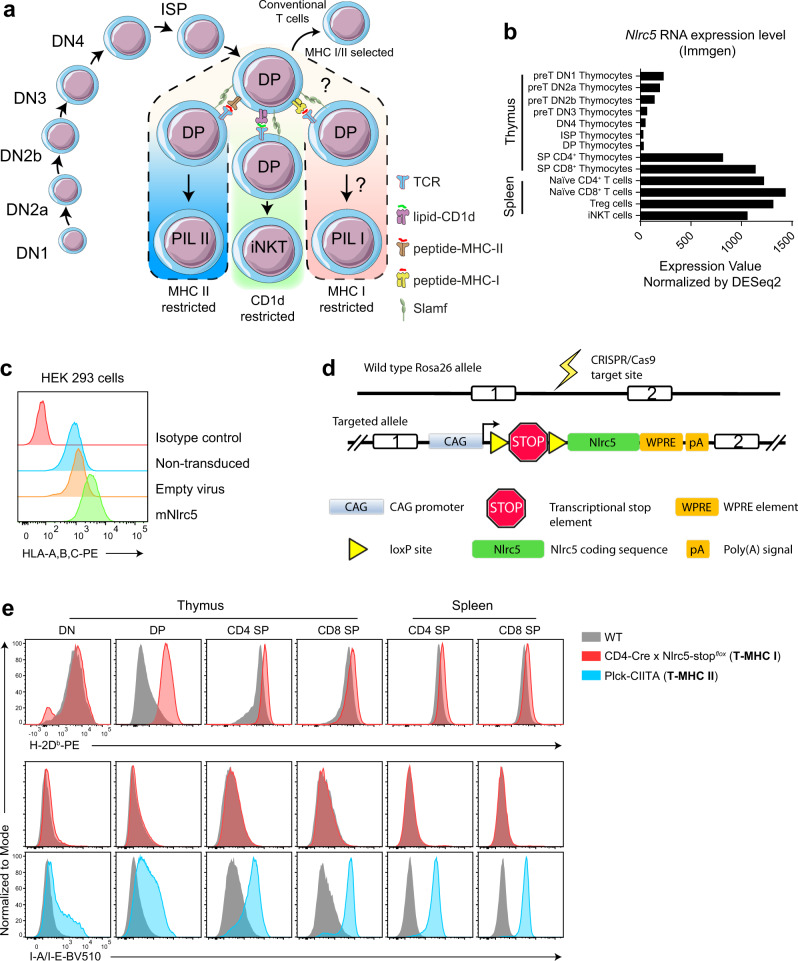
An inducible *Nlrc5* (CITA) approach to force DP thymocytes to express MHC Class I molecules. **a** Schematic representation of positive selection of innate-like T cells on DP thymocytes. **b** mRNA expression level of murine *Nlrc5* in different T-cell subsets from B6 WT mice. Data were obtained from ImmGen. **c** Flow cytometry analysis of HEK 293 cells transduced with lentivirus expression vector encoding the murine *Nlrc5* coding sequence (CDS) or with empty virus as a control. As additional controls, non-transduced cells and isotype control staining are shown. Cells are analyzed 48 h post transduction. **d** Structure of the wild-type (WT) Rosa26 and the targeted allele of the transgenic mouse (Nlrc5-stop^flox^). **e** Flow cytometry evaluation of MHC I and MHC II expression on T cells from WT, CD4-Cre × Nlrc5-stop^flox^ (T-MHC I), and Plck-CIITA (T-MHC II) transgenic mice. Data are representative of five independent experiments, *n* ≥ 5 mice per experimental group. Source data are provided as a Source Data file.

It was previously shown that the expression of MHC II on DP thymocytes promotes the development of MHC II-restricted thymocyte-selected CD4 T cells (T-CD4) with an NKT-like phenotype^21,22,24,29^. Therefore, we hypothesized that MHC I expression on DP thymocytes could have a similar effect by selecting MHC I-restricted NKT-like T cells (Fig. 1a). Normally, murine thymocytes at the DP stage of their development do not express classical MHC I or MHC II molecules^19^. In search of a suitable approach to drive stable MHC I expression in DP, we selected the transcription factor Nlrc5 (also known as CITA) as a top candidate with such potential function. Nlrc5 is crucial for transcription not only of MHC I genes but also of components necessary for MHC I processing and peptide loading such as β2m, Tap1, and Lmp2^25,28^. In addition, *Nlrc5* expression is heavily repressed at the DP thymocyte stage (Fig. 1b data from ImmGen^30^). To test whether Nlrc5 could drive higher MHC I expression in vitro, we cloned the coding sequence (CDS) of the murine *Nlrc5* gene into a lentiviral expression vector. Indeed, transduced HEK 293 cell samples showed that Nlrc5 was sufficient to drive higher MHC I expression in comparison to control samples (Fig. 1c).

To test the role of *Nlrc5* in vivo, we generated an inducible transgenic knock-in mouse where Nlrc5 expression can be upregulated in a tissue-specific manner, in cells that express Cre recombinase. In brief, a construct with a loxP-flanked stop cassette in front of the murine *Nlrc5* CDS was inserted into the *Rosa26* locus (Fig. 1d). This design allows *Nlrc5* expression once Cre recombinase is present in the cell. This mouse line was crossed to the CD4-Cre mouse initiating Cre expression at the DP stage. We observed that MHC I (H-2Db and H-2Kb) was highly expressed on DP thymocytes in these mice, compared to controls, which normally do not express it (Fig. 1e and Supplementary Fig. 1a). Single-positive (SP) thymocytes and splenocytes, which normally express high levels of MHC I, displayed only slightly higher levels in these mice. Of note, MHC II expression was not affected by the *Nlrc5* transgene (Fig. 1e).

The non-classical MHC Ib alleles that present peptides, Qa-1, Qa-2, and H2-M3, showed the same expression pattern on thymocytes as the classical MHC Ia alleles H2-Kb and H2-Db (i.e., low on DP thymocytes) (Supplementary Fig. 1d). *Nlrc5* overexpression led to their upregulation as well (Supplementary Fig. 1b). In contrast, gene expression of non-peptide presenting molecules CD1d and MR1 is high in DP thymocytes (Supplementary Fig. 1e) and was not altered by overexpression of *Nlrc5* (Supplementary Fig. 1c). This suggests that peptide presentation through MHC Ia/Ib molecules, in particular, is absent in cortical DP thymocytes due to the absence of *Nlrc5* expression, whereas lipid and metabolite presentation are preserved.

As an additional control, we obtained plck-CIITA transgenic mice^21^, which overexpress MHC II on T cells starting from the DP thymocyte stage (Fig. 1e). In the following sections, both strains were analyzed in parallel. For simplicity, we refer to CD4-Cre/Nlrc5-stop^*flox*^ as “T-MHC I” mouse and pLck-CIITA as “T-MHC II” mouse.

### MHC I expression on DP thymocytes resulted in an increase in PIL T cells

**Fig. 2:**
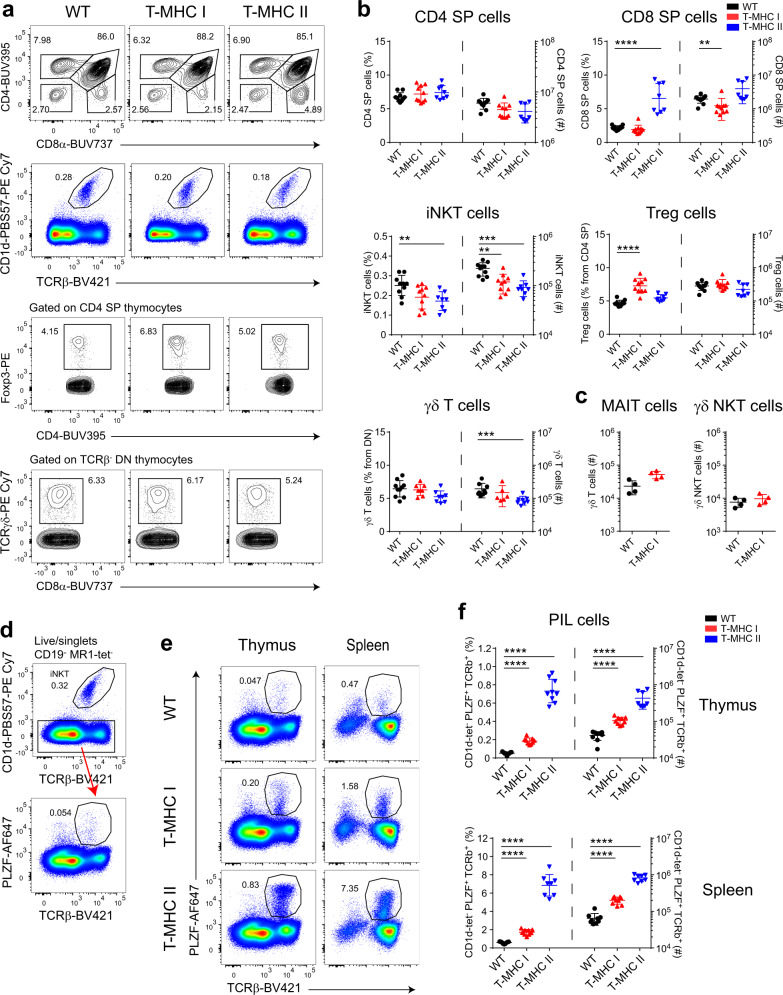
Expression of MHC I on DP thymocytes increased PLZF^+^ innate-like T cells (PILs) and decreased NKT cell numbers. **a** Representative flow cytometry plots of total thymocytes from WT, T-MHC I, and T-MHC II mice. **b** Data quantification according to the gating strategy displayed in **a**. Cell frequencies are plotted on the left axis and numbers on the right axis. **c** Total cell counts of MAIT cells and γδNKT cells in the thymus of WT and T-MHC I mice. **d** Representative plots of flow cytometry strategy for the identification of PIL cells in WT thymus. **e** Representative flow cytometry plots of staining for PIL cells comparing WT with T-MHC I and T-MHC II mice from the thymus and spleen. **f** Frequency (plotted on the left axis) and number (plotted on the right axis) of PIL cells from WT, T-MHC I, and T-MHC II mice defined by the flow cytometry strategy depicted in **d**. **b**, **c**, **f** Each point represents one animal: *n* = 10 animals per group (WT and T-MHC I groups) and *n* = 8 animals (T-MHC II group) in **b**, *n* = 4 animals per group in **c** and *n* = 9 animals per group in **f**. Data are representative of five in **a**, **b** and eight in **d**–**f** independent experiments. One experiment was performed in **c**. Unpaired two-tailed Mann–Whitney test was performed in **b**, **c**, **f**); *p* ≥ 0.01 are not depicted, **p < 0.01, ***p < 0.001, and ****p < 0.0001. Data are *p*resented as mean values ± SD. Source data are provided as a Source Data file.

The thymus architecture and the general subset distribution in the thymus showed no striking alterations between wild type (WT) and T-MHC I littermates (Fig. 2a–c and Supplementary Fig. 2). There were no significant differences in the frequency of DN, DP, CD4 SP T cells, γδ T cells, or γδ NKT cells (defined as PLZF^+^ γδ T cells). T-MHC II mice show an increased CD8 SP frequency due to the induction of “memory phenotype” CD8 T cells, as previously described^31^. In contrast, T-MHC I mice showed, if anything, a slight reduction in CD8 SP T-cell number and frequency, and a small increase in MAIT cell number (Fig. 2a–c and gating in Supplementary Fig. 3a–c). iNKT cells were approximately twofold reduced in number and frequency in both T-MHC I and T-MHC II mice. The reduction in iNKT cell number and frequency was even more prominent in the spleen and the same reduction trend was present in the liver as well (Supplementary Fig. 4a, b). There were no significant differences in the MAIT cell and γδ NKT cell number in the spleen (Supplementary Fig. 4d, e). Surprisingly, T_reg_ cell frequency was not affected in T-MHC II mice but was increased in the thymus and spleen of T-MHC I mice. In the spleen, this difference was also statistically significant in cell number (Supplementary Fig. 4b lower panel). Overall, the T-MHC I transgenic mouse did not show major lymphocyte development alterations, except for a modest increase in T_reg_ and a decrease in iNKT cell frequency and numbers.

Next, we sought to determine whether cells with NKT-like phenotype were expanded in the T-MHC I transgenic mouse similar to that seen with T-MHC II mice (Fig. 1a). PLZF is the major transcription factor endowing innate-like features to the iNKT cell lineage^14^. Therefore, we established a gating strategy that allowed identification of peptide-specific PIL T cells, by gating on TCRβ^+^PLZF^+^ cells and excluding other subsets known to express PLZF, such as iNKT cells, γδ T cells, and MAIT cells (Fig. 2d). With this strategy, we identified a small fraction of PIL T cells present in WT littermates, increasing by fourfold in T-MHC I mice (Fig. 2e, f). Nonetheless, this change was less robust than the 10–15-fold increase of PIL T cells in the T-MHC II.

### PIL T-cell expansion is SAP dependent and not mediated by Nlrc5 in a cell-intrinsic way

**Fig. 3:**
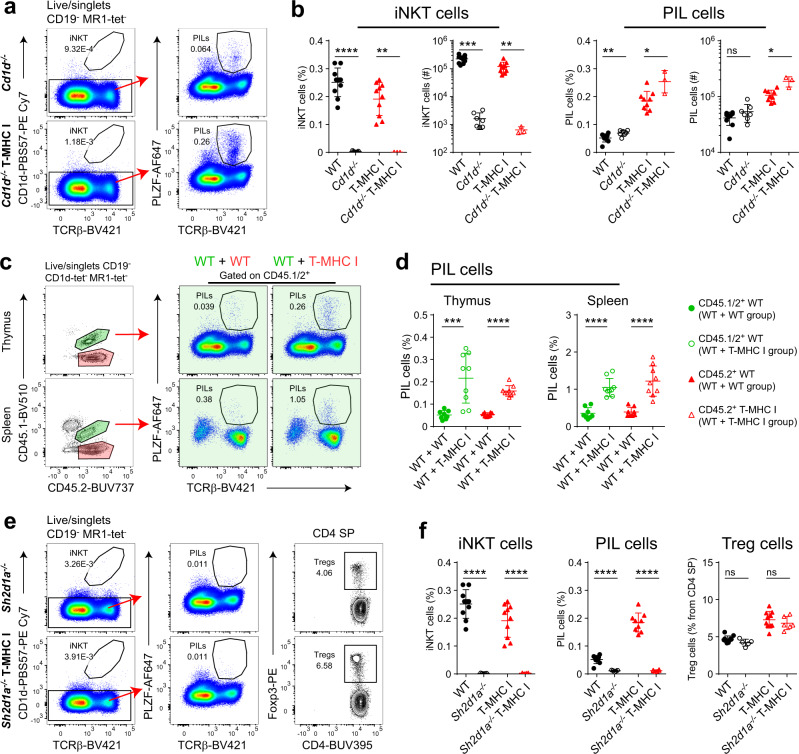
PIL T cells are SAP dependent and require MHC I in a cell-extrinsic manner. **a** Representative flow cytometry plots of total thymocytes from *Cd1d*^−/−^ and *Cd1d*^−/−^ T-MHC I mice. **b** Thymic iNKT and PIL cell frequency and number comparison between WT and *Cd1d*^−/−^, and T-MHC I and *Cd1d*^−/−^ T-MHC I mice. **c** Representative flow cytometry plots from a set of unequal bone marrow (BM) chimeric mice 8 weeks post transplantation. WT CD45.1^+^ mice were lethally irradiated and transplanted with a mix of WT CD45.1/2^+^ and T-MHC I CD45.2^+^ bone marrow at a ratio of 1 : 10. This experimental group is depicted as WT + T-MHC I group. In the control group, mice were transplanted with a mix of WT CD45.1/2^+^ and WT CD45.2^+^ bone marrow at a ratio of 1 : 10. This experimental group is depicted as WT + WT group. Shown are representative flow cytometry plots displaying the gating strategy from WT + WT group (in the left two panels). In the right four panels (in green) are shown representative plots displaying PIL cell frequencies (gated on WT CD45.1/2^+^) from the WT + WT group (on the left) and WT + T-MHC I group (on the right). PIL cell frequency evaluation is shown in **d**. **e** Representative flow cytometry plots of total thymocytes from *Sh2d1a*^−^^*/−*^ (SAP deficient) and *Sh2d1a*^*−/−*^ T-MHC I mice. iNKT, PIL, and T_reg_ cell frequency are shown in **f**. **b**, **d**, **f** Each point represents one animal: *n* = 10 animals per group (WT and T-MHC I groups), *n* = 7 animals (*CD1d*^*−/−*^ group), *n* = 3 animals (*CD1d*^*−/−*^ T-MHC I group), *n* = 8 animals (WT + WT group), *n* = 9 animals (WT + T-MHC I group), *n* = 5 animals (*Sh2d1a*^*−/−*^ group), and *n* = 6 animals (*Sh2d1a*^*−/−*^ T-MHC I group). Data are representative of four in **a**, **b**, **e**, **f** and two in **c**, **d** independent experiments. Unpaired two-tailed Mann–Whitney test was performed in **b**, **d** and an unpaired two-tailed Student’s *t*-test was performed in **f**; ns, not significant (*p* ≥ 0.05), *p < 0.05, **p < 0.01, ***p < 0.001, and ****p < 0.0001. Data are presented as mean values ± SD. Source data are provided as a Source Data file.

Even though we did not observe upregulation of surface CD1d in response to Nlrc5 expression in DP thymocytes, it is formally possible that PIL T cells are CD1d dependent. Therefore, we crossed T-MHC I mice to *Cd1d*^−/−^ mice. Deficiency of CD1d abrogated iNKT cell development, as expected, but if anything, increased PIL T-cell numbers in T-MHC I mice (Fig. 3a, b). Thus, most PIL T cells are likely MHC Ia/Ib restricted, as their numbers are dependent on MHC I expression in DP thymocytes, yet not dependent on CD1d.

Next, we investigated whether MHC I was required on neighboring DP thymocytes for PIL T-cell development, or if it is due to an unknown cell-intrinsic effect of Nlrc5 overexpression. To this end, we established a set of unequal BM chimeric mice, where WT recipients were lethally irradiated and transplanted with a mix of WT and T-MHC I BM at a ratio 1 : 10 from different congenic mice. With this setup, 90% of the neighboring thymocytes would express MHC I and is thus capable of inducing PIL T-cell selection in WT progenitors. A control group received a mix of WT and WT BM from different congenic mice. Indeed, 8 weeks post transplantation, a significant increase in PIL T cells was present among WT BM-derived cells when neighboring thymocytes were expressing MHC I, in comparison to the control group (Fig. 3c, d). A significant increase in PIL T-cell frequency was present also in the spleen. Therefore, this result disfavors the possibility that forced expression of Nlrc5 promotes PILs development in a cell-intrinsic way and confirms the hypothesis that PIL T cells expand because of MHC I upregulation on neighboring DP thymocytes. Notably, the frequency of T_reg_ cells did not increase among the WT BM-derived cells but was only observed among the T-MHC I-derived cells (Supplementary Fig. 5a, b). This indicates that the observed expansion of T_reg_ cells in the T-MHC I mouse is mediated by Nlrc5 expression in a cell-intrinsic way.

A crucial requirement for iNKT cell development is a triggering of SLAM-SAP signaling pathways during agonist selection^13^. Therefore, we sought to determine whether SAP deficiency has the same impact on PIL T cell as on iNKT cell development. Expectedly, lack of SAP completely abrogated iNKT cell development. It also abrogated the generation of PIL T cells in both the thymus (Fig. 3e, f) and spleen (Supplementary Fig. 5c, d). This indicates that PIL T cells and iNKT cells employ similar signaling pathways during their selection and development.

### PIL T cells are found in the same effector subsets as iNKT cells

**Fig. 4:**
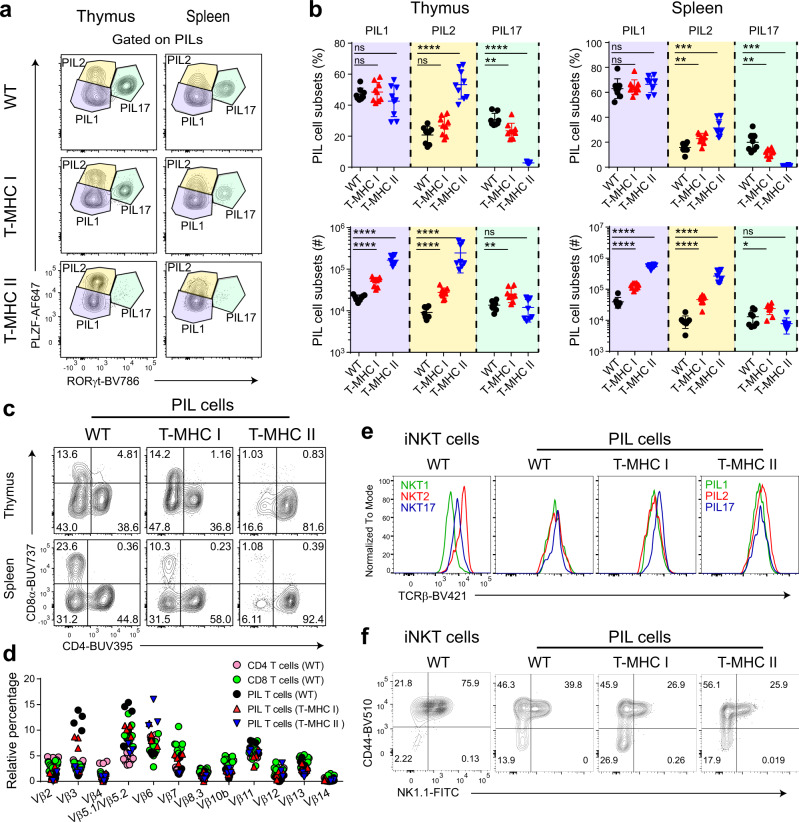
Both MHC I and MHC II induced PIL T cells exist in three major subsets, similar to NKT cells. **a** Akin to iNKT cells, PIL T cells segregate into three subsets defined by expression pattern of transcription factors PLZF and RORγt. Shown are representative flow cytometry plots comparing PIL T-cell subsets from WT, T-MHC I, and T-MHC II mice. **b** Comparisons of PIL T-cell subset frequencies (upper row) and numbers (lower row). **c** Representative flow cytometry plots of staining for CD4 and CD8α on PIL cells from WT, T-MHC I, and T-MHC II mice. **d** Flow cytometry assessment of the TCR Vβ chain repertoire of thymic PIL T cells from WT, T-MHC I, and T-MHC II mice in comparison to conventional T cells. **e** Exemplary plots showing staining for TCRβ on iNKT cell subsets compared to PIL T-cell subsets from WT, T-MHC I, and T-MHC II mice. All analyzed animals are F1 generation with BALB/c mice (further characterized in Fig. 6). **f** CD44 and NK1.1 expression pattern on (from left to right) iNKT, WT PIL, T-MHC I PIL, and T-MHC II PIL T cells. **b** Each point represents one animal: *n* = 8 animals per group (WT and T-MHC II groups) and *n* = 9 animals (T-MHC I group). Data are representative of seven in **a**–**c** and three in **d**–**f** independent experiments. Unpaired two-tailed Mann–Whitney test was performed in **b**; ns, not significant (*p* ≥ 0.05), *p < 0.05, **p < 0.01, ***p < 0.001, and ****p < 0.0001. Data are presented as mean values ± SD. Source data are provided as a Source Data file.

Thymic iNKT cells exist in three well-defined effector subsets (iNKT1, 2, and 17), analogous to peripherally activated helper T cells^32^. Using transcription factor staining, we found that PIL T cells also segregate into three similar subsets, which we name as PIL1 (PLZF^lo^Tbet^+^RORγt^-^), PIL2 (PLZF^hi^RORγt^-^), and PIL17 (PLZF^int^RORγt^+^) cells (Fig. 4a). The expression of NK1.1 on PIL1, CD4 on PIL2, and CD138 on PIL17 cells was also similar to that of iNKT cell subsets (Supplementary Fig. 6a). Hence, this is suggestive that each PIL fraction might exhibit similar functional properties to the corresponding iNKT cell subset. Several independent studies have shown that the three NKT subsets have quite distinct gene expression programs^33–35^. Indeed, expression of a large panel of functionally relevant molecules, including CD122, CXCR3, CD69, PD1, CCR6, and CD25 was similar among PIL and iNKT subsets (Supplementary Fig. 6b), as was the production of the cytokines interferon-γ (IFNγ), interleukin (IL)-4, and IL-17A after in vitro stimulation (Supplementary Fig. 6c)^32,33^. Taken together, these data infer that analogous transcriptional and functional programs operate in PIL and iNKT cells.

Notably, both MHC I- and MHC II-restricted PIL T cells were differentiated into three subsets (Fig. 4a, b). However, MHC II-restricted PIL T cells in T-MHC II animals showed a strong skewing toward the PIL2 effector phenotype, at the expense of PIL17. This is consistent with the well-described overproduction of IL-4 by PLZF^+^ T-CD4s in pLck-CIITA mice^31^. However, the three subsets were present in similar proportions in WT and T-MHC I mice (Fig. 4a, b).

As mentioned above, PIL T cells might be phenotypically and functionally similar to iNKT cells. Yet, in contrast to iNKT cells, they are selected on classical MHC molecules. Therefore, we inspected their TCR co-receptor expression and TCR Vβ chain usage. As previously reported, PIL T cells developing in T-MHC II mice were found to be largely CD4 SP with a small fraction of DN cells (Fig. 4c). Surprisingly, PIL T cells in T-MHC I mice did not exclusively express CD8, but segregated into CD4 SP, CD8 SP, and DN, similar to those in WT mice, suggesting that PIL T cells in WT mice may be more closely related to MHC I-restricted cells (Fig. 4c). Although we did not observe a severe bias in TCR Vβ chain usage by PIL T cells, there was a noticeable increase in frequency of Vβ3^+^ and Vβ5.1/Vβ5.2^+^ cells within the PIL T-cell pool from WT and T-MHC I mice. In addition, a small increase in frequency of Vβ6^+^ cells was present within the PIL T cells from T-MHC II mice (Fig. 4d).

It was previously shown that thymic iNKT cell subsets display different levels of TCR on their surface, a trait that is more prominent on the BALB/c background^33,36^. TCR levels are highest on NKT2 cells, lowest on NKT1 cells, and intermediate on NKT17 cells. Moreover, these differences in TCR expression levels, and thus the presumed signaling strength, are reported to be pivotal for iNKT cell subset commitment and differentiation^37–39^. However, surface TCR did not differ significantly between PIL T-cell subsets (Fig. 4e), suggesting that factors other than TCR expression level may dictate subset commitment and differentiation in these cells.

Historical studies in the field of iNKT cell biology used CD44 and NK1.1 as markers to assess iNKT cell maturity and stage of development^40^. Using these markers, a higher proportion of PIL T cells were found to be CD44^low^ compared to iNKT cells, indicating a less mature stage (Fig. 4f).

### Memory phenotype CD8 T-cell development requires CD4 co-receptor engagement in PIL T cells

**Fig. 5:**
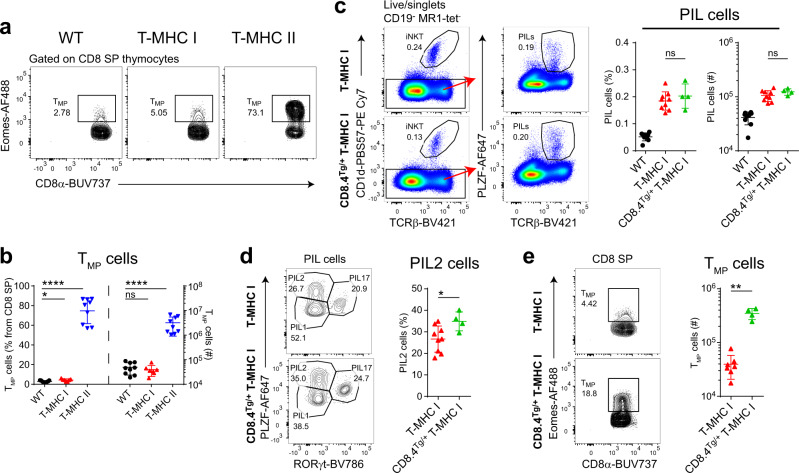
MHC II, but not MHC I, drives type 2 cytokine production in PIL T cells and induces memory phenotype CD8 T (T_MP_)-cell development. **a** Representative flow cytometry plots of intracellular staining for Eomes on CD8 SP thymocytes from WT, T-MHC I, and T-MHC II mice. **b** Number (plotted on the right axis) and frequency (plotted on the left axis) of CD8 T_MP_ cells among CD8 SP cells in the thymus from WT, T-MHC I, and T-MHC II mice, defined by the gating strategy shown in **a**. **c** Representative flow cytometry plots of thymocytes comparing PIL T-cell frequency from T-MHC I mice with CD8.4^Tg/+^ T-MHC I mice. Shown are summary evaluations for PIL T-cell number and frequency (right two panels). **d** Representative flow cytometry plots and summary evaluation for frequency of PIL2 T-cell subset among thymocytes from T-MHC I and CD8.4^Tg/+^ T-MHC I mice. Both groups are compared to WT mice. **e** Exemplarity flow cytometry plots of CD8 T_MP_ cell frequency among CD8 SP cells in the thymus from T-MHC I mice with CD8.4^Tg/+^ T-MHC I mice (left two panels) and summary evaluation of CD8 T_MP_ cell number (right panel). Each point represents one animal: *n* = 9 animals per group (WT and T-MHC II groups) in **a**, **b**, *n* = 9 animals (T-MHC I group) in **c**, **d**, *n* = 7 animals (T-MHC I group) in **b**, **e**, and *n* = 4 animals (CD8.4^Tg/+^ T-MHC I group) in **c**–**e**. Data are representative of 7 in **a**, **b** and 2 in **c**–**e** independent experiments. Unpaired two-tailed Mann–Whitney test was performed in **b**–**e**; ns, not significant (*p* ≥ 0.05), *p < 0.05, **p < 0.01, and ****p < 0.0001. Data are presented as mean values ± SD. Source data are provided as a Source Data file.

As previously reported, the frequency of CD8 SP T cells was increased in the thymus of T-MHC II mice (Fig. 2b)^21,22,31^. This was shown to be caused by IL-4 produced by PIL T cells (called T-CD4 cells in that report), which mediates development of memory phenotype CD8 T (T_MP_) cells through the induction of eomesodermin (Eomes)^41^. Thus, a large proportion of CD8 SP T cells in T-MHC II mice express Eomes (Fig. 5a) and have a memory phenotype^31^. To our surprise, there was no increase in Eomes^+^ CD8 T_MP_ in T-MHC I mice (Fig. 5b). As interaction of the TCR with MHC II can invoke CD4 co-receptor signaling, we reasoned that the induction of IL-4 production by PIL T cells and, consequently, the increase in CD8 T_MP_ cells might be the result of CD4 co-receptor signaling. To test this hypothesis, we crossed T-MHC I mice with CD8.4 transgenic mice. In these mice, the cytoplasmic tail of the endogenous *Cd8a* gene was substituted with the cytoplasmic tail of CD4^42^. The CD8.4 transgene did not cause an increase in the total number of PIL T cells in T-MHC I mice (Fig. 5c), but it shifted the proportion of PIL T cells in favor of PLZF^hi^ PIL2 cells (Fig. 5d), which produce IL-4. Consistent with this, the number of CD8 T_MP_ was markedly increased in CD8.4 T-MHC I mice (Fig. 5e). A similar trend was seen in the spleen (Supplementary Fig. 7). Thus, the particular co-receptor involved in sensing MHC ligands on DP thymocytes influences effector subset differentiation of PIL T cells and has secondary effects of CD8 T_MP_ development.

### PIL T cells compete with iNKT cells for a cellular niche within the thymus

**Fig. 6:**
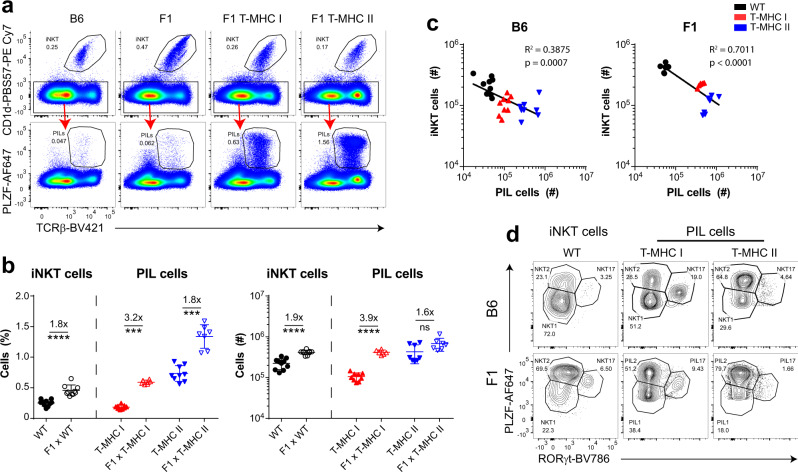
PIL T cells compete with iNKT cells. **a** B6 WT, T-MHC I, and T-MHC II mice were crossed to BALB/c mice and F1 generation littermates (labeled as F1, F1 T-MHC I, and F1 T-MHC II) were analyzed by flow cytometry for frequency of iNKT and PIL T cells in the thymus. **b** Summary evaluation of iNKT and PIL T-cell frequency (left panel) and number (right panel) from WT, T-MHC I, and T-MHC II mice on B6 and F1 background. **c** Inverse correlation between number of iNKT cells and the number of PIL T cells in the thymus from WT (black dots), T-MHC I (red triangles), and T-MHC II mice (blue inverse triangles) on B6 (left panel) and F1 (right panel) background. **d** Shown are representative flow cytometry plots comparing iNKT and PIL T-cell subsets from WT, T-MHC I, and T-MHC II mice on B6 (upper row) and F1 (lower row) background. Each point represents one animal: *n* = 10 animals per group (WT and T-MHC I groups), *n* = 8 animals (T-MHC II group), *n* = 9 animals (F1 WT group), *n* = 6 animals (F1 T-MHC I group), and *n* = 7 animals (F1 T-MHC II group) in **a**–**d**. Data are representative of seven independent experiments. Unpaired two-tailed Mann–Whitney test was performed in **b**; ns, not significant (*p* ≥ 0.05), ***p < 0.001, and ****p < 0.0001. *R*^2^-values and *p*-values in **c** were calculated by fitting nonlinear regression and performing a Goodness-of-Fit test and extra-sum-of-squares F test. Data are presented as mean values ± SD. Source data are provided as a Source Data file.

Similar to what have been previously reported with T-MHC II mouse^43^, we observed a two- to threefold reduction in the number of lipid-specific iNKT cells in mice with increased numbers of PIL T cells due to MHC I or MHC II expression on DP thymocytes (Fig. 2b). Of note, the pooled total cell count of PILs + iNKT cells was not significantly different between WT and T-MHC I mice (data not shown). As both peptide and lipid-specific T cells require SAP signaling from the surface SLAM family co-receptors to develop into PIL T cells or iNKT cells, respectively, this suggests that the two cell types may be in competition with each other for a cellular niche within the thymus. To further test this notion, we examined B6xBALB/c F1 mice, which have a slightly larger (twofold) iNKT cell niche^32,44^. If a similar niche regulates PIL T cells, then we might expect more PIL T cells in F1 mice. Indeed, both the portion and number of PIL T cells in T-MHC I and T-MHC II mice were higher in animals on the F1 background compared to the B6 background (Fig. 6a, b and Supplementary Fig. 8a, b) inferring an existence of a bigger niche for PIL T-cell development on the F1 background. We observed an inverse relationship between the number of iNKT cells and PIL T cells in both B6 and F1 mice (Fig. 6c and Supplementary Fig. 8c). Taken together, with the expansion of PIL T cells in mice that lack iNKT cells (Fig. 3b), these data strongly suggest that PIL T cells and iNKT cells compete for the same cellular niche. As an aside, the reported skewing of iNKT cells towards the PFZF^hi^ NKT2 subset in F1 mice^32^ was also observed in PIL T cells (Fig. 6d and Supplementary Fig. 8d). This suggests that the same factors control iNKT and PIL T-cell effector subset skewing in different strains, which are more likely to be environmental cytokines, cell-intrinsic factors, or co-stimulatory molecules rather than specific self-antigens.

## Discussion

Here we describe an *Nlrc5*-transgenic mouse (T-MHC I) allowing cell subset-specific expression of *Nlrc5*, which is a central regulator of the expression and function of peptide presenting MHC I genes. Consequently, our data show that forced expression of *Nlrc5* led to upregulation of MHC I on DP thymocytes. To the best of our knowledge, this is the first transgenic mouse model allowing cell subset-specific MHC I upregulation without altering MHC II regulation. Hence, this mouse line can serve as a powerful tool in future studies where stable MHC I expression is sought. Notably, *Nlrc5* expression increased the surface level of some non-classical MHC Ib molecules such as Qa-1 and Qa-2 (peptide-presenting molecules) but did not affect CD1d and MR1 (non-peptide presenting molecules). Therefore, in our mouse model, we assume enhanced self-peptide MHC Ia/Ib presentation by DP thymocytes renders them capable of steering peptide-specific T cells towards an innate-like developmental pathway. Although we do not provide a direct proof for the nature of the antigens that PIL T cells recognize, previous studies showed that DP thymocytes could not select PIL T cells in a foreign antigen-specific TCR transgenic T-MHC II mouse model^22^. This implies that PIL T-cell selection is self-antigen specific and requires presentation by an opposing DP thymocyte. In addition, a subsequent study showed that PIL T cells from T-MHC II mice have a highly diverse TCR repertoire, implying they may recognize a variety of self-antigens^45^. Taken together, it is reasonable to consider that PIL T cells could be selected on endogenous peptide self-antigens presented by DP thymocytes, much similar to iNKT cells selected on an endogenous lipid self-antigen presented by DP thymocytes^3,9–11^.

Although DP thymocytes do not normally express classical MHC Ia molecules, a small fraction of PIL T cells were already present in WT mice. Yet, we show that most of these cells are not CD1d or MR1 restricted. Therefore, it remains unclear how these innate-like cells were selected. Interestingly, the small number of PIL T cells present in WT mice more closely resemble PIL T cells from T-MHC I mice than those from T-MHC II mice. This implies that PIL T cells in WT mice might be restricted to MHC Ib molecules, which are found to be still expressed, although at a low level, on DP thymocyte (i.e., H2-M3 and Qa-1). Indeed, it was previously shown that peptide-specific MHC Ib-restricted T cells could be selected on TECs or HCs^46^. However, those cells acquired an innate-like phenotype only when they are selected on HCs^18,46^. In addition, this process was shown to be SAP dependent^47^. Therefore, it is plausible that PIL T cells in WT mice are MHC Ib restricted.

Although MHC I expression on DP thymocytes led to an expansion of PIL T cells, the magnitude of this expansion was lower than that in the T-MHC II mouse. Further, the increased PIL T-cell frequency in the T-MHC I mouse did not result in a notable expansion of CD8 T_MP_ cells, implying functional difference between PILs selected in T-MHC I and T-MHC II mice. Of note, mature murine iNKT cells do not express CD8αβ. In fact, several studies have previously shown that forced expression of CD8αβ is detrimental for iNKT cell development and leads to profound reduction in iNKT cell numbers^7,48^. Subsequently, another study showed that the silencing of CD8 expression in developing iNKT cells is mediated by the transcription factor Th-POK, whose expression is required for functional maturity of iNKT cells^49^. Therefore, the co-receptor choice seems to govern the functional phenotype of PIL cells as well. As a result, despite being selected on MHC I molecules, PIL cells developing in T-MHC I mice might be downregulating CD8 expression post selection due to initiation of the innate-like developmental program and expression of Th-POK. In addition, previous reports suggest that TCR signal strength seems to regulate iNKT cell subset commitment and differentiation^37,38,50^. Stronger TCR signal intensities favor iNKT2 cell development and a lower strength preferentially guides into the iNKT1 cell pathway. Moreover, CD4 co-receptor signaling intensity is higher in comparison to CD8^51–53^ and presumably favors PIL2 cell expansion in T-MHC II mice. Therefore, increasing co-receptor signaling intensity by crossing T-MHC I to the CD8.4 mouse^42^ may have increased the frequency of PIL2 cells and expansion of CD8 T_MP_ cells.

There are several studies suggesting a possible involvement of *Nlrc5* expression in pro-inflammatory and type I IFN responses^54,55^. Although we did not observe any major alterations of conventional T-cell development, forced *Nlrc5* expression in our mouse model caused an increase in T_reg_ cell number and frequency. Moreover, here we show that this expansion was mediated in a cell-intrinsic manner by *Nlrc5* and it was not because of MHC I expression on neighboring DP thymocytes. Interestingly, Nlrc5 is debatably described as a positive or negative modulator of nuclear factor-κB (NF-κB) signaling^56–58^. NF-κb signaling is also known to be crucial for T_reg_ cell development and function^59,60^. Taking this into consideration, it is possible that *Nlrc5* expression at the DP stage might influence T_reg_ cell selection and development by modulating NF-κB signaling. Further studies will be needed to identify the precise mechanism behind Nlrc5 involvement in T_reg_ cell biology.

Lastly, we provide several lines of evidence supporting the existance of a narrow niche for innate-like T-cell development. First, iNKT cell number and frequency were reduced in both T-MHC I and T-MHC II mice. Second, this inverse correlation between iNKT cell and PIL T-cell numbers was even more profound on a B6xBALB/c F1 background where the niche for iNKT cell development is larger. In addition, CD1d-deficient T-MHC I mice showed an increase in PIL T cells. Innate-like T cells might compete between each other for abundance of cytokines, activation ligands, or could directly inhibit each other’s development and survival via unknown mechanisms. Of note, the local abundance of IL-7, IL-15, and IL-25^61–63^, in the thymus, are crucial factors implicated in iNKT cell development, survival, and terminal subset maturation. Thus, higher PIL T-cell numbers might alter the bioavailability of these cytokines in the thymus. Yet, MAIT cells and γδ NKT cells did not display a reduction trend in the T-MHC I mouse. Therefore, this is suggestive that PIL T cells and iNKT cells probably compete for the same activation ligands, presumably members of the SLAMF receptors.

MHC class I molecules are some of the most widely expressed genes in the body. In contrast to MHC class II molecules, which are expressed primarily by professional antigen presenting cells, MHC class I is generally thought to be expressed by all nucleated cells. Based on this, the absence of MHC I in DP thymocytes is remarkable. The fact that we observed a reduction of lipid-specific iNKT cells when peptide-presenting MHC alleles were forced to be expressed in DP thymocytes suggests an evolutionary rationale for the absence of MHC I in DP thymocytes—to facilitate the development of innate-like T cells that recognize non-peptide ligands. Indeed, iNKT cells do recognize non-peptide ligands such as lipids^64^. Given that these cells also are semi-invariant means that their rearrangements occur relatively rarely, despite the great numeric abundance of these cells in the body^10^. Thus, the thymus appears to have a mechanism whereby the development of innate-like T cells from rare TCR gene rearrangements is favored.

## Methods

### Mice

C57BL/6NCrl (B6) and B6.SJL-PtprcaPepcb/BoyCrCrl (CD45.1) mice were obtained from Charles River (via the National Cancer Institute). BALB/cByJ, B6.129S6-Sh2d1atm1Pls/J (SAP^−/−^), C57BL/6-Tg(Lck-CIITA)16Spark/J (Plck-CIITA), and B6.129S6-Del(3Cd1d2-Cd1d1)1Sbp/J (CD1d^−/−^) mice were purchased from the Jackson Laboratories. CD8.4 transgenic mice were kindly provided by Dr. Alfred Singer (NCI/CCR, Bethesda). All animals were maintained under specific pathogen-free conditions at the University of Minnesota. All animals used in this study were 6–10 weeks old at the time of analysis. All experimental procedures were approved by the institutional animal care and use committee at the University of Minnesota (IACUC 1706-34889A and 1709-35136A).

### Generation of Nlrc5-stop^flox^ transgenic mouse

The Nlrc5-stop^flox^ transgenic mice were generated by the insertion of a *CAG-LoxP-STOP-LoxP-Nlrc5-WPRE-pA* construct into mouse *Rosa26* locus through the CRISPR/Cas9 system with C57BL/6J embryo. The generated mice were screened and further verified for the correct insertion by sequencing and Southern blotting. The generation of Nlrc5-stopflox mice was conducted by Biocytogen.

### BM chimeras

BM cells were isolated from the femur and tibia of B6 CD45.1/2^+^, B6 CD45.2^+^, and T-MHC I CD45.2^+^ donor mice. Following isolation, cells were counted and washed twice in cold phosphate-buffered saline (PBS). B6 CD45.1/2^+^ cells (5 × 10^5^) mixed with 4.5 × 10^6^ T-MHC I CD45.2^+^ cells were mixed and transferred intravenously into lethally irradiated (900 rads) CD45.1 recipient mice. The control group received 5 × 10^5^ B6 CD45.1/2^+^ cells mixed with 4.5 × 10^6^ B6 CD45.2^+^ cells. Mice were killed 8 weeks post reconstitution when the spleen and thymi were collected for flow cytometry analysis.

### Flow cytometry

Organs were collected and single-cell suspensions were prepared on ice in fluorescence-activated cell sorting buffer (PBS/3% fetal calf serum). All surface stainings were performed for 30 min on ice. Intracellular detection of cytokines and transcription factors were done using the Foxp3/Transcription Factor Staining Buffer Kit (Tonbo Biosciences, TNB-0607-KIT), following the protocol provided by the manufacturer. Antibodies used (clone name, dilution factor) were as follows: anti-mouse CD4 BUV395 (GK1.5, 1 : 400), anti-mouse CD8a BUV737 (53-6.7, 1 : 400), anti-mouse Qa-1(b) PE (6A8.6F10.1A6, 1 : 100), anti-mouse CD1d PE (1B1, 1 : 100), anti-mouse PLZF AF647 (R17-809, 1 : 200), anti-mouse CD45.2 BUV737 (104, 1 : 200), anti-mouse RORγt BV786 (Q31-378, 1 : 400), anti-mouse/rat CD44 BV510 (IM7, 1 : 200), anti-mouse IL-4 APC (11B11, 1 : 100), anti-mouse Vβ2 TCR FITC (B20.6, 1 : 100), anti-mouse Vβ3 TCR FITC (KJ25, 1 : 100), anti-mouse Vβ4 TCR FITC (KT4, 1 : 100), anti-mouse Vβ5.1/Vβ5.2 TCR FITC (MR9-4, 1 : 100), anti-mouse Vβ6 TCR FITC (RR4-7, 1 : 100), anti-mouse Vβ7 TCR FITC (TR310, 1 : 100), anti-mouse Vβ8.3 TCR FITC (1B3.3, 1 :100), anti-mouse Vβ10b TCR FITC (B21.5, 1:100), anti-mouse Vβ11 TCR FITC (RR3-15 1 : 100), anti-mouse Vβ12 TCR FITC (MR11-1, 1 : 100), anti-mouse Vβ13 TCR FITC (MR12-3, 1 : 100), and anti-mouse Vβ14 TCR FITC (14-2, 1 : 100) all from BD Bioscience; anti-human HLA-A,B,C PE (W6/32, 1 : 200), anti-mouse I-A/I-E BV510 (M5/114.15.2), anti-mouse H-2Ld/H-2Db PE (28-14-8, 1 : 100), anti-mouse H-2Kb PE (AF6-88.5, 1 : 100), anti-mouse Qa-2 FITC (695H1-9-9, 1 : 100), anti-human/mouse/rat MR1 APC (26.5, 1 : 100), anti-mouse TCRb BV421 (H57-597, 1 : 100), anti-mouse TCR γ/δ PE-Cy7 (GL3, 1 : 100), anti-mouse CD45.1 BV510 (A20, 1 : 200), anti-mouse NK1.1 FITC (PK136, 1 : 100), anti-mouse CD138 PE (281-2, 1 : 200), anti-mouse CD122 PE (TM-β1, 1 : 100), anti-mouse CXCR3 FITC (CXCR3-173, 1 : 100), anti-mouse CD196 (CCR6, 1 : 100) PE (29-2L17), anti-mouse CD25 PE (PC61, 1 : 100), and anti-mouse IL-17A PE (TC11-18H10.1, 1 : 100) all from BioLegend; and anti-mouse/rat Foxp3 PE (FJK-16s, 1 : 200), anti-mouse PLZF AF488 (Mags.21F7), anti-mouse CD69 PE (H1.2F3, 1 : 200), anti-mouse CD19 PerCP-Cy5.5 (eBio1D3, 1 : 200), anti-mouse CD279 (PD-1) PE (J43, 1 : 200), anti-mouse IFNγ PE (XMG1.2, 1 : 100), and anti-mouse Eomes AF488 (Dan11mag, 1 : 100) all from eBioscience. CD1d tertramer loaded with PBS57 (analog of a-galactosylceramide) and MR1 tetramer loaded with 5-OP-RU were provided by the tetramer facility of the US National Institutes of Health. Flow cytometric analysis was performed on LSR Fortessa (BD Biosciences) using FACSDiva software (version 6.1.3, BD Biosciences) and data analysis was done using FlowJo software (Treestar).

### Analysis of intracellular cytokines

Total thymocytes were plated at a density of 1 × 10^6^ ml^−1^ and incubated at 37 °C for 4 h in the presence of Cell Stimulation Cocktail (eBioscience, 00-4975-93) and Protein Transport Inhibitor Cocktail (eBioscience, 00-4980-93) in RPMI 1640/10% fetal bovine serum followed by cytokine detection by intracellular staining.

### Enrichment of MAIT cells

Single-cell suspensions were prepared from the thymus or spleen and incubated with PE-MR1–5-OP-RU tetramer for 30 min at ambient temperature. Following incubation, anti-PE microbeads (Miltenyi) were used for immunomagnetic enrichment following the manufacturer’s instructions.

### Immunofluorescence

Thymi were fixed with 4% paraformaldehyde overnight, transferred to 30% sucrose solution for 24 h, and snap frozen in optimum cutting temperature compound. Sections (7 μm) were blocked with 5% bovine serum albumin and Fc block (anti-CD16/CD32; 2.4G2, Tonbo Biosciences) in dilution 1 : 100 for 1 h at room temperature (RT) prior to staining. The sections were incubated with Rabbit-anti-b5t in dilution 1 : 200 (MBL International) and fluorescein-labeled Ulex europaeus agglutinin I (UEA-I) (Vector Laboratories) at 4 °C overnight, followed by Goat-anti-Rabbit-AF555 in dilution 1 : 500 for 1 h at RT (Thermo Fisher Scientific). Sections were next stained with 4′,6-diamidino-2-phenylindole and mounted using ProLong antifade mounting medium (Life Technologies). Images were acquired using a Leica DM6000B epifluorescent microscope.

### Cloning mNlrc5 and lentiviral transduction experiments

The full-length CDS of murine Nlrc5 was cloned through PCR using primers Nlrc5-Frw: 5′-GATTCTAGAGCCACCATGGACGCTGAGAGCATCC-3′ and Nlrc5-Rev: 5′-GCAATCGATTTAATTAATCAAAGAGTCTGCTGGTCAGTG-3′ with cDNA from B6 CD8 T-cell splenocytes. Nlrc5 cDNA was further subcloned into lentiviral expression vector lentiCas9-EGFP and further verified by DNA sequencing. Nlrc5 expression lentivirus (lentiNlrc5-EGFP) was generated as previously described^65^. As a control, an empty construct (lenti-EGFP) was generated encoding EGFP (enhanced green fluorescent protein) as a marker for transduction efficacy. Viral supernatants were used to transduce HEK-293T cell. HEK-293T cells were analyzed for MHC I expression by flow cytometry 48 h post transduction.

### Statistics and reproducibility

Statistical analysis was performed using GraphPad Prism v7 and v8 Software. An unpaired, two-tailed Mann–Whitney *U*-test was done when group sample size *n* was ≤4 in one or both of the groups, which were compared. For groups with sample size *n* > 4, a Kolmogorov–Smirnov test was used to check for the distribution normality of the data points in each of the groups to be compared. When both groups showed a normal distribution, then an unpaired two-tailed Student’s *t*-tests was performed. When one or both of the compared groups showed non-normal distribution, then an unpaired two-tailed Mann–Whitney *U*-test was used as indicated in the figure legends. Samples are shown with medians with error bars showing the SD. *p*-values of <0.05 (*), <0.01 (**), <0.001 (***), or <0.0001 (****) indicated significant differences between groups. All attempts at replicating experiments were successful.

### Reporting summary

Further information on research design is available in the Nature Research Reporting Summary linked to this article.

## Supplementary information

Supplementary Information

Reporting summary

## Data Availability

The data that support the findings of this study are available from the corresponding author upon reasonable request. Source data are provided with this paper.

## Source data

Source Data
