# Supplementary material for: Classical MHC expression by DP thymocytes impairs the selection of non-classical MHC restricted innate-like T cells
Source: Nat Commun. 2021 Apr 16;12:2308. doi: 10.1038/s41467-021-22589-z (PMC8052364; doi:10.1038/s41467-021-22589-z)
Supplement: Supplementary file 1 — Supplementary Information [file 41467_2021_22589_MOESM1_ESM.pdf]

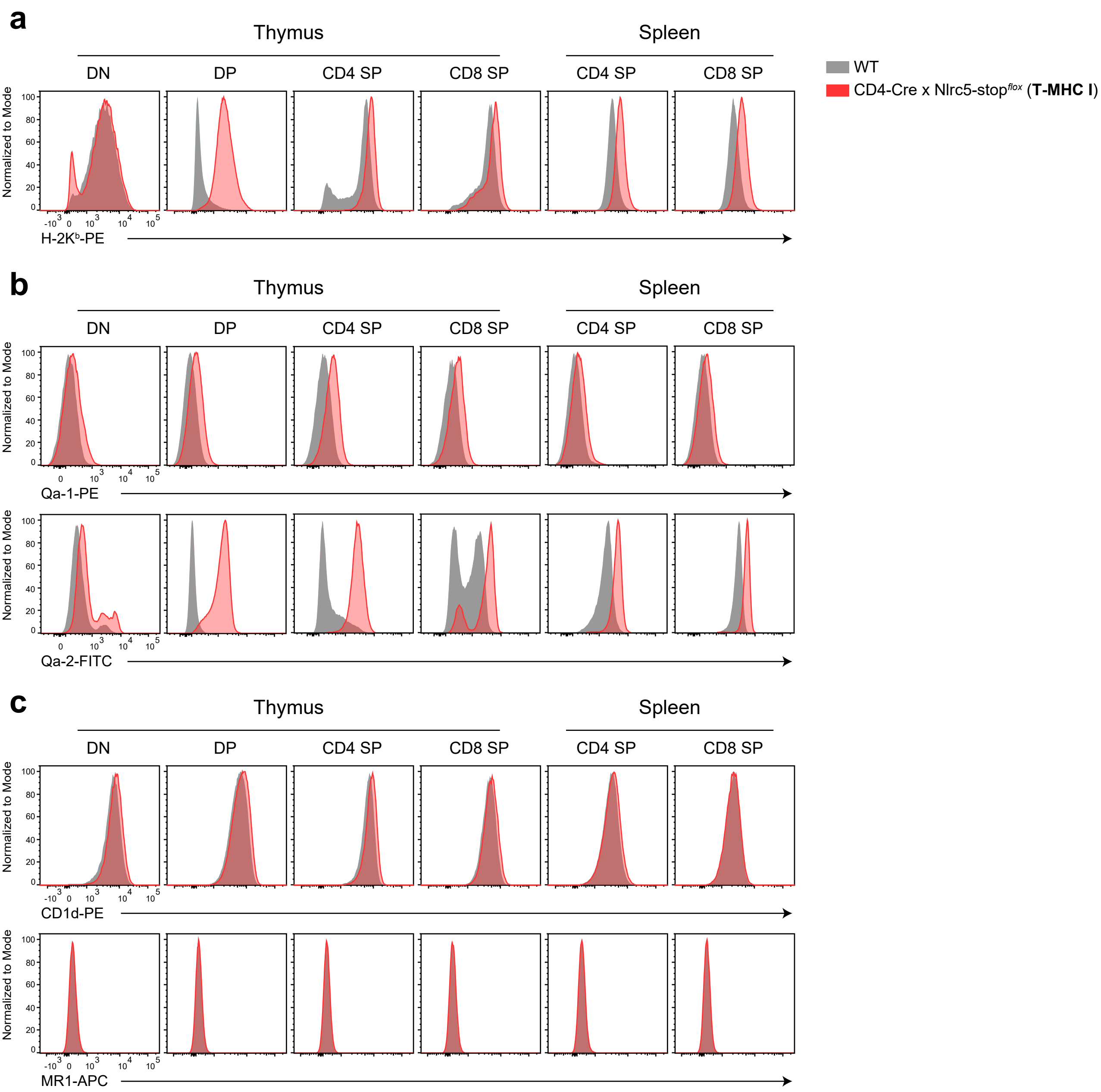
a
Thymus
Spleen
DN
DP
CD4 SP
CD8 SP
CD4 SP
CD8 SP
WT
CD4-Cre x Nlrc5-stop^{flox} (**T-MHC I**)
Normalized to Mode
H-2Kᵇ-PE
b
Thymus
Spleen
DN
DP
CD4 SP
CD8 SP
CD4 SP
CD8 SP
Normalized to Mode
Qa-1-PE
Normalized to Mode
Qa-2-FITC
c
Thymus
Spleen
DN
DP
CD4 SP
CD8 SP
CD4 SP
CD8 SP
Normalized to Mode
CD1d-PE
Normalized to Mode
MR1-APC

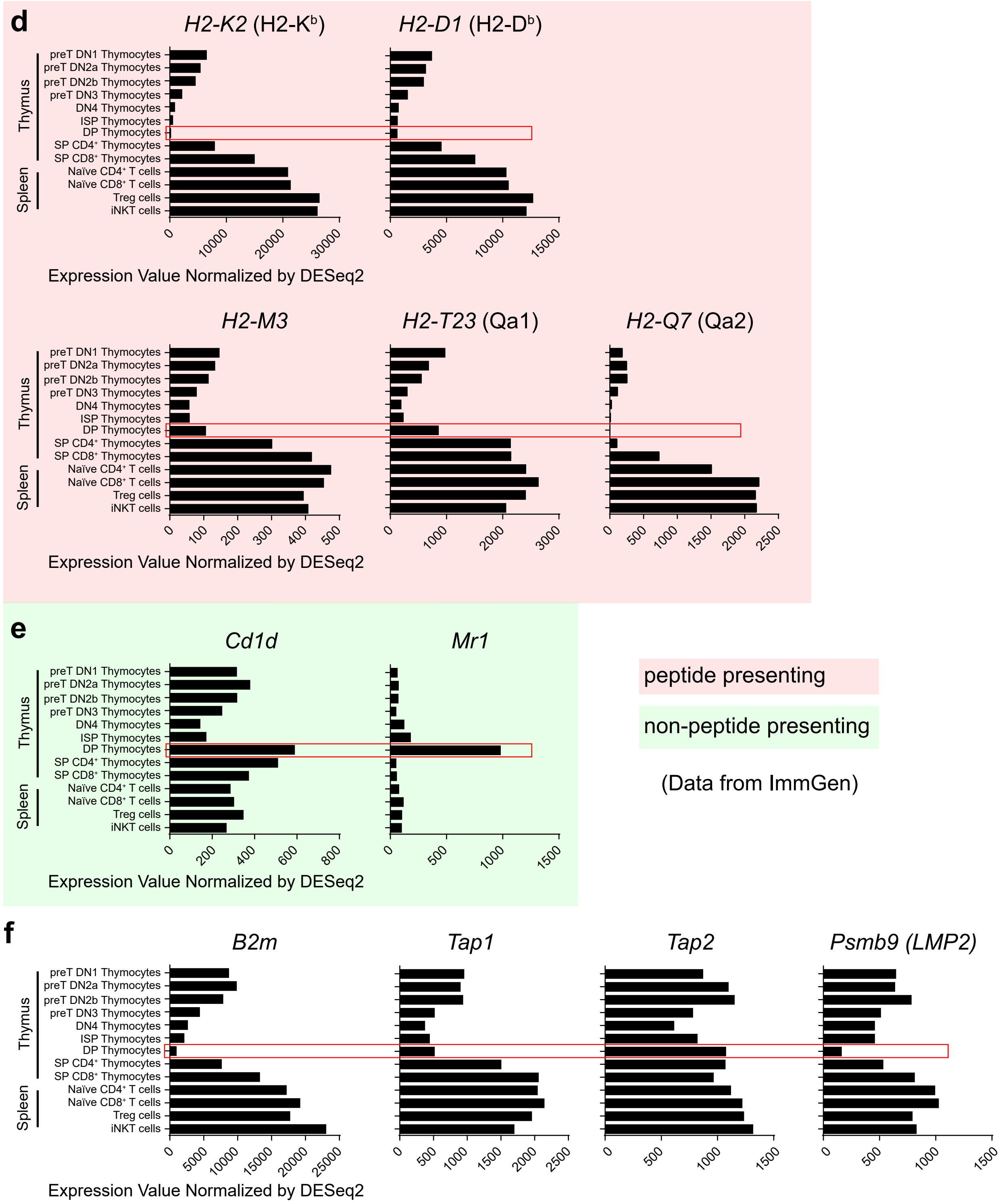

Supplementary Fig. 1. Expression profile of MHC class I molecules in WT and T-MHC I mouse. a-c, Expression pattern of peptide presenting classical MHC Ia molecule (H-2Kb) in (**a**), peptide presenting non-classical MHC Ib molecules (Qa-1 and Qa-2) in (**b**) and non-peptide presenting non-classical MHC Ib molecules (CD1d and MR1) in (**c**) on T cells from WT and CD4-Cre x Nlrc5-stop^{flox} (T-MHC I) transgenic mouse. **d-f,** mRNA expression profile of peptide presenting and non-peptide presenting MHC I molecules. Shown are mRNA expression levels in T cells of peptide presenting MHC I alleles in (**d**), non-peptide presenting MHC I alleles in (**e**) and of components necessary for MHC I processing and peptide loading in (**f**). Data are representative of 5 in (a,b) and 3 in (**c**) independent experiments with n=5 animals per each experimental group in (a-b) and n=3 animals per each experimental group in (**c**). Source data are provided as a Source Data file.

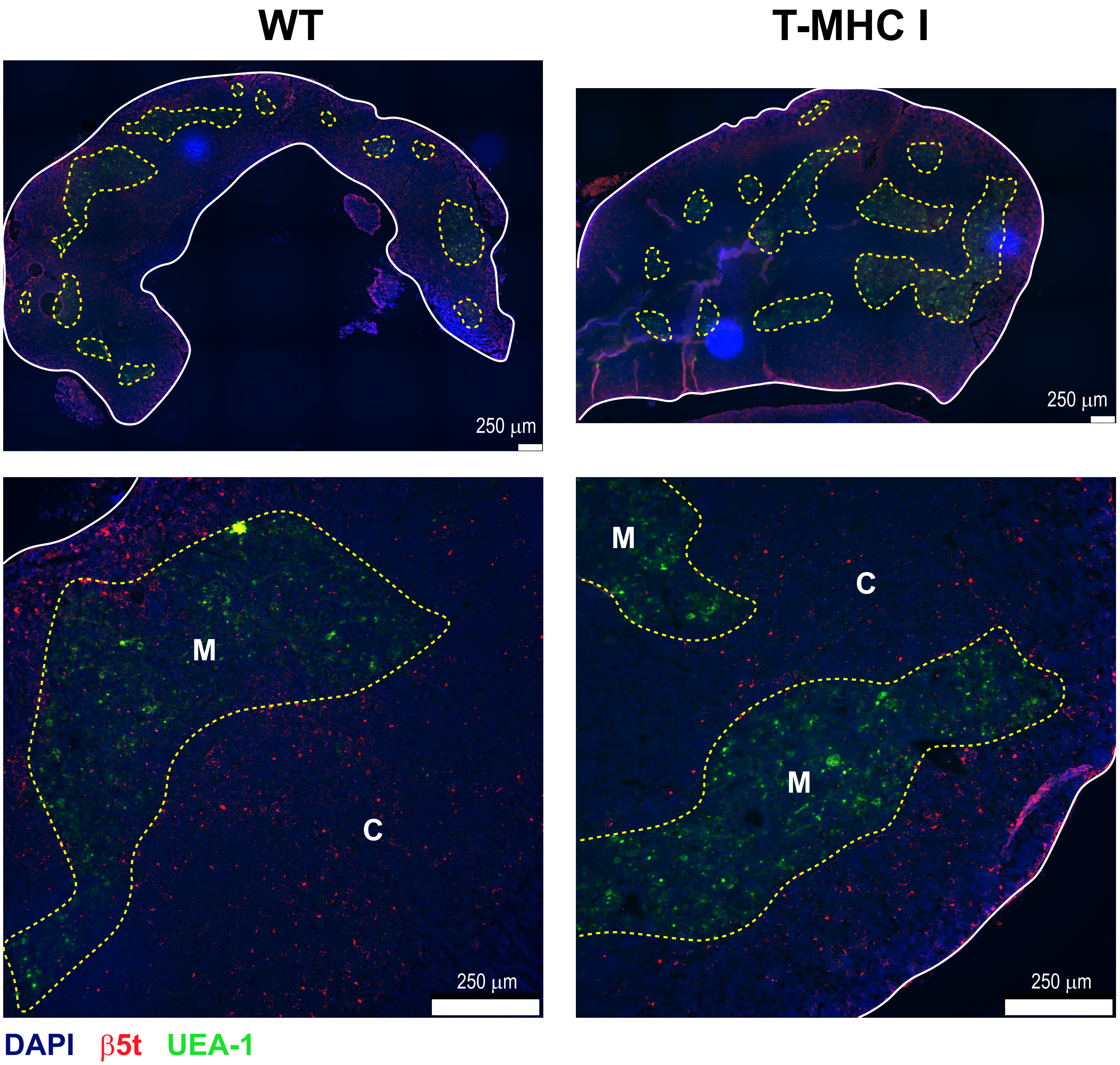

Supplementary Fig. 2. Immunofluorescence microscopy of thymic sections of WT (left two panels) and T-MHC I (right two panels) mice, stained for β5t (red), UEA-1 (green) and with DAPI (blue) used to define the thymic medulla (M) and cortex (C). The yellow dashed line outlines the thymic medulla. Data are representative of 1 experiments with two animals per genotype. Scale bar = 250 µm.

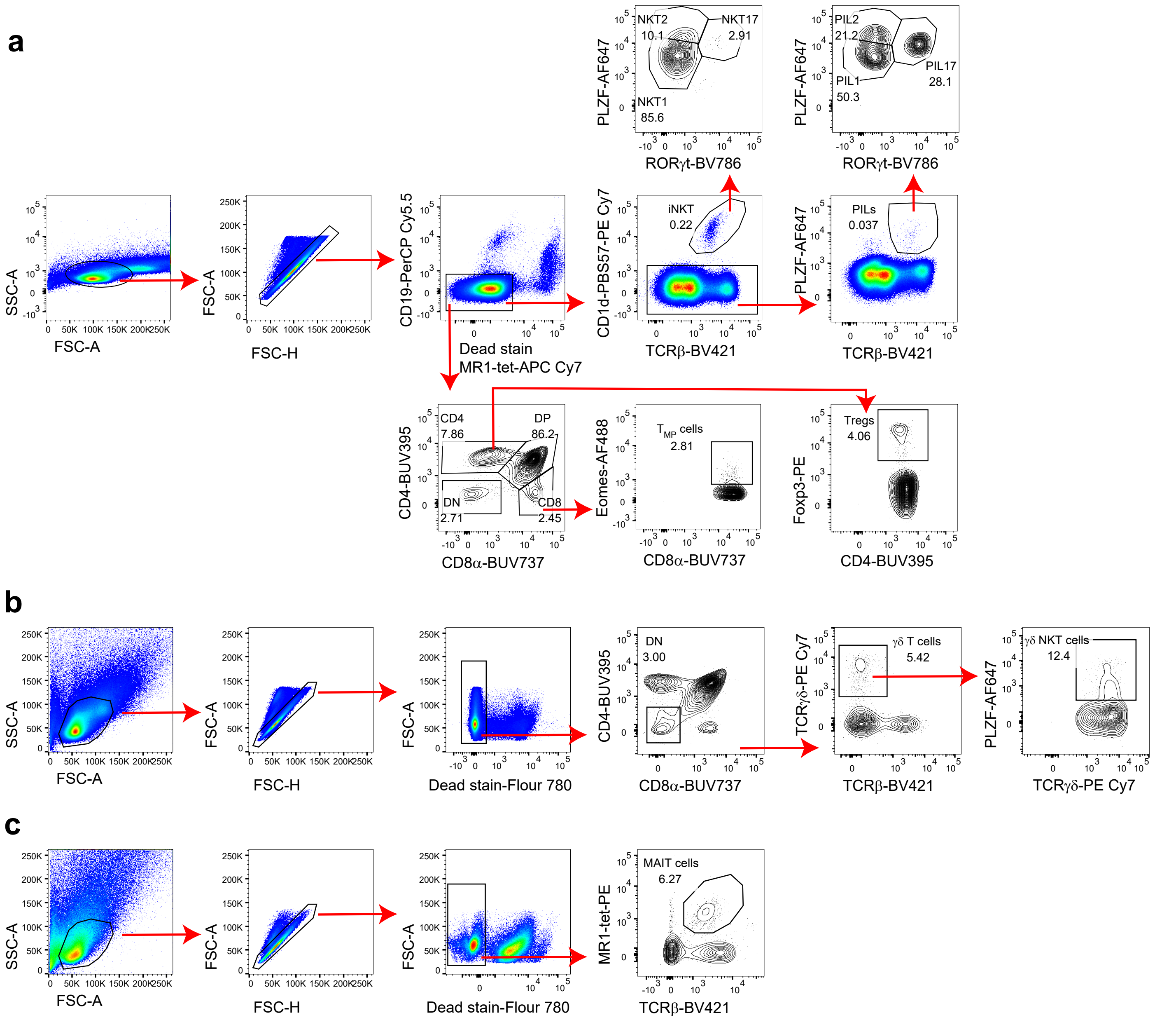

Supplementary Fig. 3. Full gating strategies used for flow cytometry analysis allowing identification of CD4 SP, CD8 SP, DP, DN, iNKT, PILs, Tregs and T_{MP} cells in (**a**), γδ T and γδ NKT cells in (**b**) and MAIT cells after tetramer pull-down in (**c**). The same gating strategies were applyed for samples from thymus, spleen and liver.

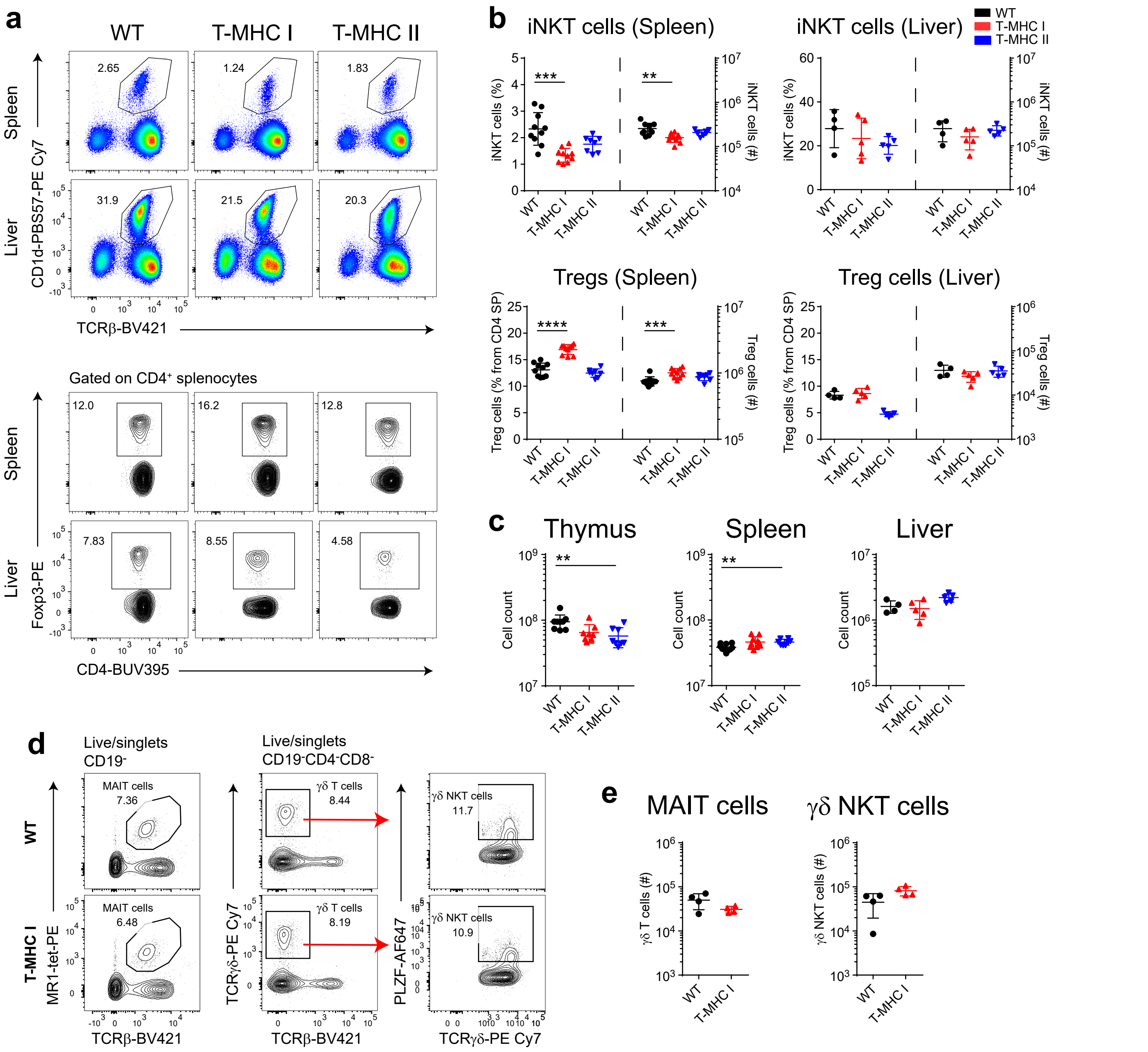

Supplementary Fig. 4. Overexpression of MHC I on DP thymocytes results in an increase in Treg cell and decreased NKT cell numbers in the spleen.

a, Representative flow cytometry plots of total cell preparations from spleen and liver of WT, T-MHC I and T-MHC II mice. **b,** Data quantification according to the gating strategy displayed in (**a**). Cell frequencies are plotted on the left axis and numbers on the right axis. **c,** Total thymocytes, splenocytes and liver cell count. **d,** Representative flow cytometry plots of total cell preparations from spleen of WT and T-MHC I mice (the right two panels) and after MR1-tetramer enrichment (the left two panels). **a-c**, Each point represents one animal: Thymus and spleen n=10 animals per group (WT and T-MHC I groups) and n=8 animals (T-MHC II group), Liver n=4 animals (WT group) and n=5 animals per group (T-MHC I and T-MHC II). **d-e**, n=4 animals per experimental group. Data are representative of 7 independent experiments in (**a-c**) and 1 experiment in (**d-e**). Unpaired two-tailed Mann–Whitney test was performed in (**b,c,e**); $p \geq 0.01$ are not depicted, $^{**}p < 0.01$, $^{***}p < 0.001$ and $^{****}p < 0.0001$. Data are presented as mean values ± SD. Source data are provided as a Source Data file.

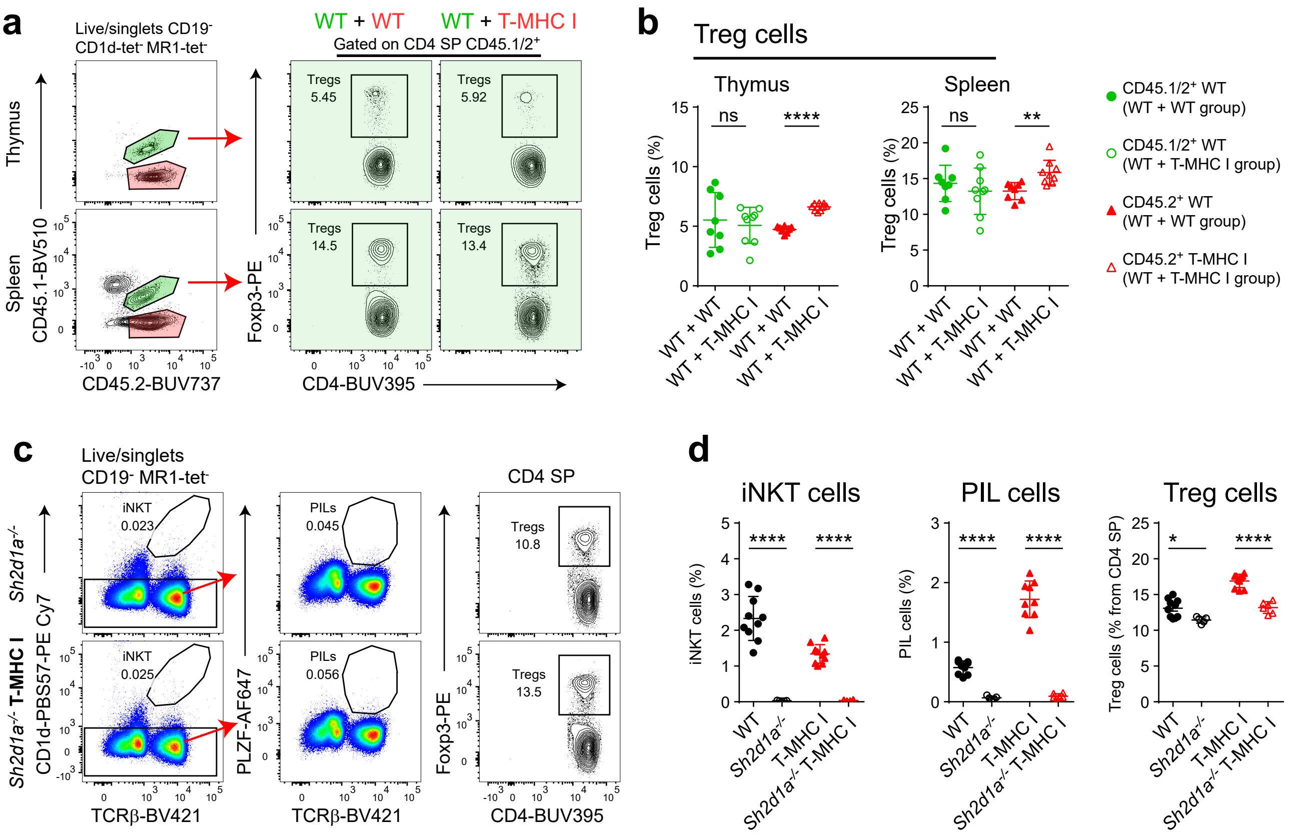

Supplementary Fig. 5. PILs are SAP dependent and Tregs require Nlrc5 in a cell-intrinsic fashon. a,b, Nlrc5 regulates Treg development in a cell-intrinsic fashon. **a,** Representative flow cytometry plots from a set of unequal bone marrow (BM) chimeric mice described in (**Fig. 3c,d**). Shown are representative flow cytometry plots gated on WT CD45.1/2$^+$ cells, displaying the frequency of Tregs cells among CD4 SP cells. Treg cells frequency evaluation is shown in (**b**). **c,d,** SAP deficient mice lack PILs in the spleen. **c,** Representative flow cytometry plots of total splenocytes from *Sh2d1a*$^{-/-}$ (SAP deficient) and *Sh2d1a*$^{-/-}$ T-MHC I mice. iNKT, PIL and Treg cell frequency are shown in (**d**). Each point represents one animal: n=8 animals (WT + WT group), n=9 animals (WT + T-MHC I group) in (**a-b**), n=10 animals per group (WT and T-MHC I groups), n=5 animals (*Sh2d1a*$^{-/-}$ group) and n=6 animals (*Sh2d1a*$^{-/-}$ T-MHC I group). Data are representative of 2 in (**a-b**) and 4 in (**c,d**) independent experiments. Unpaired two-tailed Mann–Whitney test was performed in (**b**) and an unpaired two-tailed *t*-test was performed in (**d**); ns, not significant ($p \geq 0.05$), *$p < 0.05$, **$p < 0.01$ and ****$p < 0.0001$. Data are presented as mean values ± SD. Source data are provided as a Source Data file.

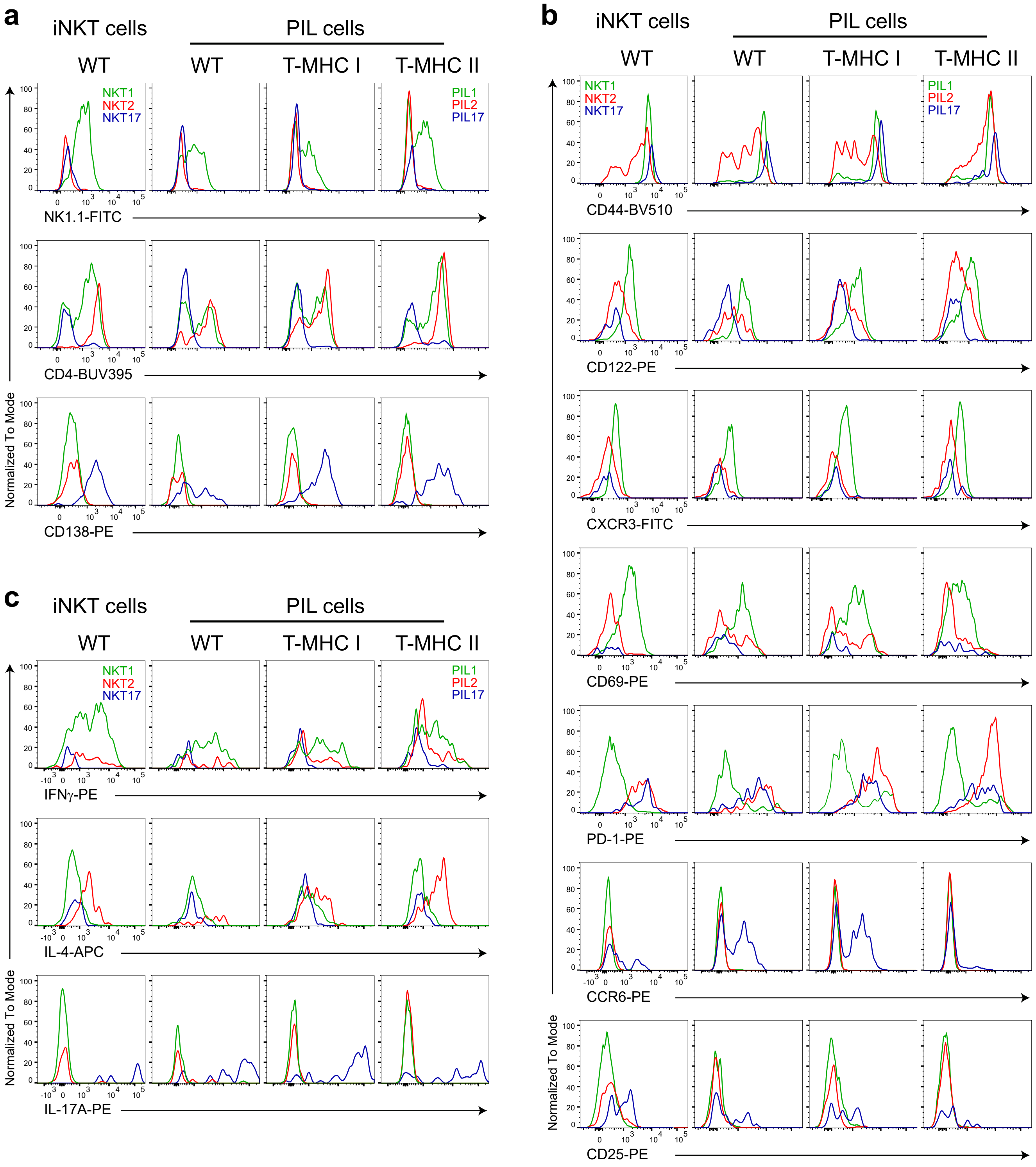

Supplementary Fig. 6. Expression pattern of iNKT cell characteristic markers and cytokines by PILs. a-c, Expression pattern of iNKT cell characteristic markers on PIL cell subsets in (**a,b**) and cytokines in (**c**). Prior cytokine detection, cells were stimulated with cell stimulation cocktail (PMA/ionomycin) *in vitro* in the presence of protein transport inhibitor cocktail (Brefeldin A/Monensin). Data are representative of: n=4 animals per each genotype in (**a,b**) and n=3 animals per each genotype in (**c**).Data are representative of 3 independent experiments.

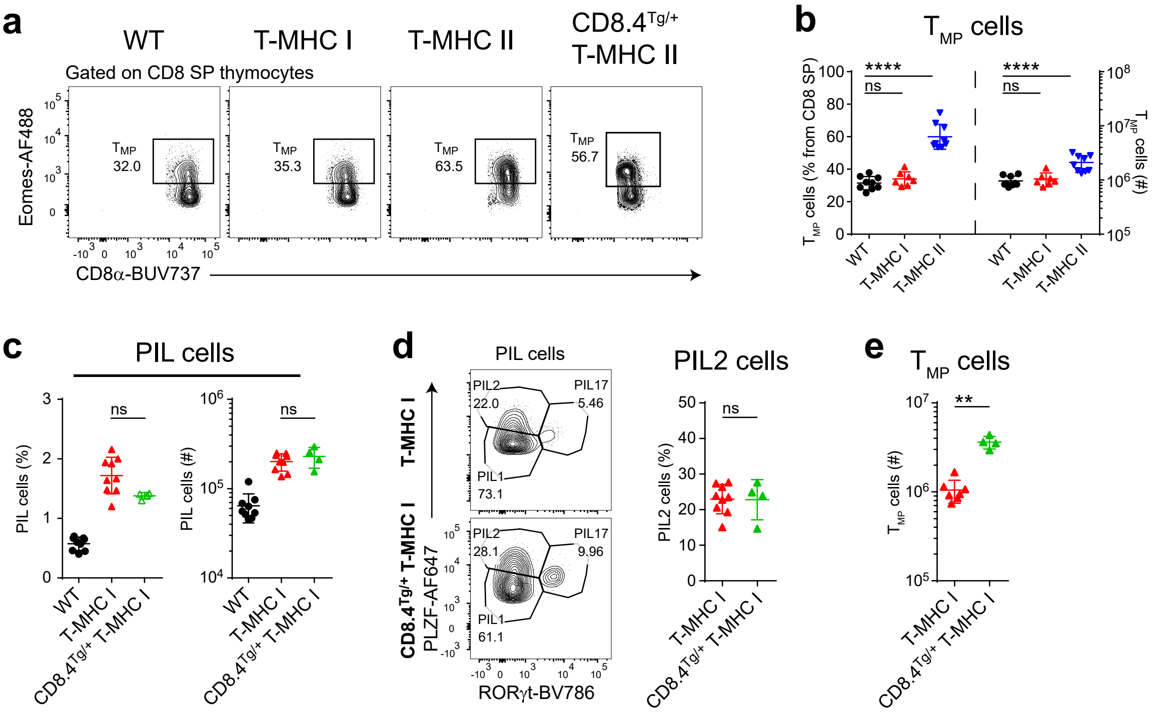

Supplementary Fig. 7. Increased memory phenotype CD8 T (T_{MP}) cell fraction in spleen of CD8.4$^{Tg/+}$ T-MHC I mouse. a, Representative flow cytometry plots of intracellular staining for Eomes on CD8 SP splenocytes from WT, T-MHC I, T-MHC II and CD8.4$^{Tg/+}$/T-MHC I mice. **b,** Number (plotted on the right axis) and frequency (plotted on the left axis) of CD8 T_{MP} cells among CD8 SP cells in spleen from WT, T-MHC I and T-MHC II mice, defined by the gating strategy shown in (**a**). **c,** Summary evaluations for PIL T cell number and frequency in spleen. **d,** Representative flow cytometry plots and summary evaluation for frequency of PIL2 T cell subset among splenocytes from T-MHC I and CD8.4$^{Tg/+}$/T-MHC I mice. Both groups are compared to WT mice. **e,** Summary evaluation of CD8 T_{MP} cell number in spleen from T-MHC I and CD8.4$^{Tg/+}$/T-MHC I mice. Each point represents one animal: n=9 animals per group (WT and T-MHC II groups) in (**a,b**), n=9 animals (T-MHC I group) in (**c,d**), n=7 animals (T-MHC I group) in (**b,e**) and n=4 animals (CD8.4$^{Tg/+}$ T-MHC I group) in (**c-e**) Data are representative of 7 in (**a,b**) and 2 in (**c-e**) independent experiments. Unpaired two-tailed Mann–Whitney test was performed in (**b-e**); ns, not significant ($p \geq 0.05$),**$p < 0.01$ and ****$p < 0.0001$. Data are presented as mean values ± SD. Source data are provided as a Source Data file.

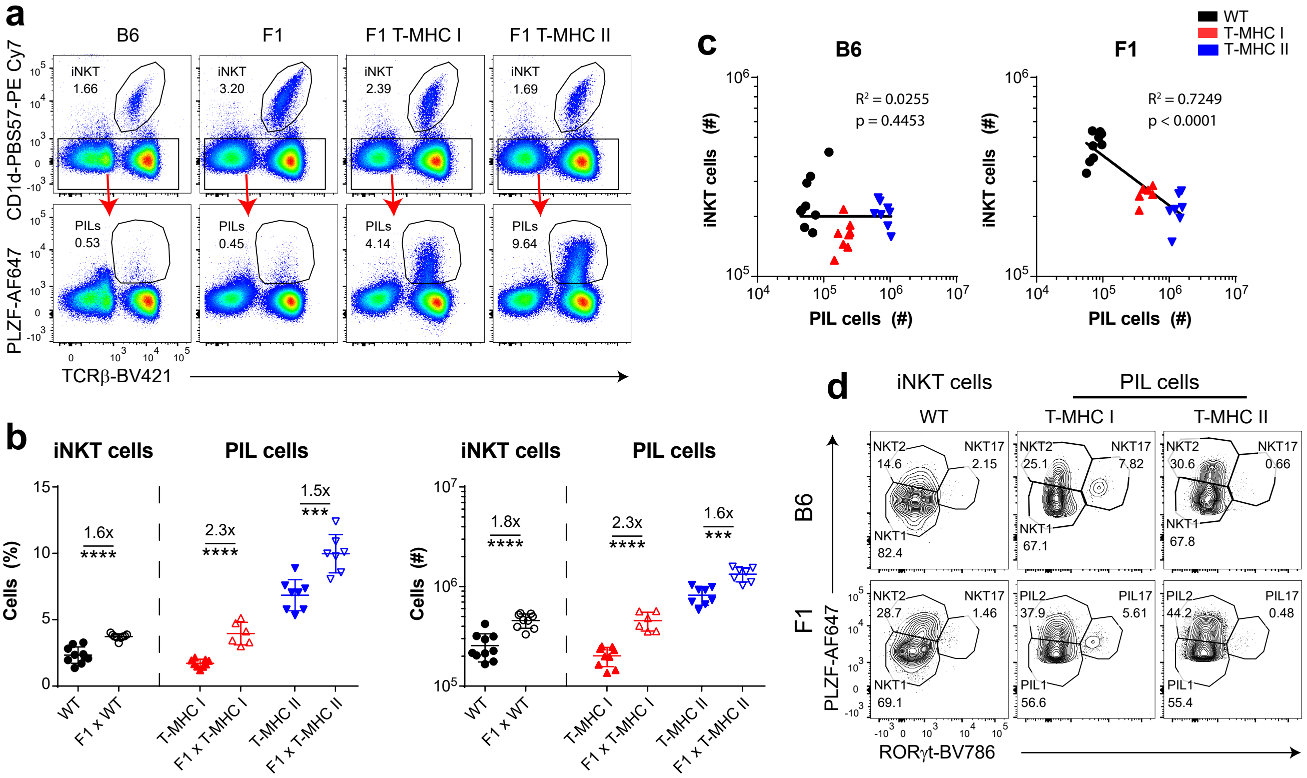

Supplementary Fig. 8. PILs compete with iNKT cells in periphery. a, B6 WT, T-MHC I and T-MHC II mice were crossed to BALB/c mice and F1 generation littermates (labeled as F1, F1 T-MHC I and F1 T-MHC II) were analyzed by flow cytometry for frequency of iNKT and PIL T cells in the spleen. **b,** Summary evaluation of iNKT and PIL T cell frequency (left panel) and number (right panel) from WT, T-MHC I and T-MHC II mice on B6 and F1 background. **c,** Inverse correlation between number of iNKT cells and the number of PIL T cells in the spleen from WT (black dots), T-MHC I (red triangles) and T-MHC II mice (blue inverse triangles) on B6 (left panel) and F1 (right panel) background. **d,** Shown are representative flow cytometry plots comparing iNKT and PIL T cell subsets from WT, T-MHC I and T-MHC II mice on B6 (upper row) and F1 (lower row) background. Each point represents one animal: n=10 animals per group (WT and T-MHC I groups), n=8 animals (T-MHC II group), n=9 animals (F1 WT group), n=6 animals (F1 T-MHC I group) and n=7 animals (F1 T-MHC II group) in (**a-d**). Data are representative of 7 independent experiments. Unpaired two-tailed t-test was performed in (**b**); ***$p < 0.001$ and ****$p < 0.0001$. R^2 values and p values in (**c**) were calculated by fitting nonlinear regression and performing a Goodness of Fit test and and extra-sum-of-squares F test. Data are presented as mean values ± SD. Source data are provided as a Source Data file.
