# Supplementary material for: Classical MHC expression by DP thymocytes impairs the selection of non-classical MHC restricted innate-like T cells
Source: Nat Commun. 2021 Apr 16;12:2308. doi: 10.1038/s41467-021-22589-z (PMC8052364; doi:10.1038/s41467-021-22589-z)
Supplement: Supplementary file 2 — Reporting summary [file 41467_2021_22589_MOESM2_ESM.pdf]

nature research

Corresponding author(s): Kristin A. Hogquist

Last updated by author(s): Dec 11, 2020

Reporting Summary

Nature Research wishes to improve the reproducibility of the work that we publish. This form provides structure for consistency and transparency in reporting. For further information on Nature Research policies, see our Editorial Policies and the Editorial Policy Checklist.

Statistics

For all statistical analyses, confirm that the following items are present in the figure legend, table legend, main text, or Methods section.

n/a	Confirmed	
☐	☒	The exact sample size (n) for each experimental group/condition, given as a discrete number and unit of measurement
☐	☒	A statement on whether measurements were taken from distinct samples or whether the same sample was measured repeatedly
☐	☒	The statistical test(s) used AND whether they are one- or two-sided<br>*Only common tests should be described solely by name; describe more complex techniques in the Methods section.*
☒	☐	A description of all covariates tested
☐	☒	A description of any assumptions or corrections, such as tests of normality and adjustment for multiple comparisons
☐	☒	A full description of the statistical parameters including central tendency (e.g. means) or other basic estimates (e.g. regression coefficient) AND variation (e.g. standard deviation) or associated estimates of uncertainty (e.g. confidence intervals)
☐	☒	For null hypothesis testing, the test statistic (e.g. F, t, r) with confidence intervals, effect sizes, degrees of freedom and P value noted<br>*Give P values as exact values whenever suitable.*
☒	☐	For Bayesian analysis, information on the choice of priors and Markov chain Monte Carlo settings
☒	☐	For hierarchical and complex designs, identification of the appropriate level for tests and full reporting of outcomes
☒	☐	Estimates of effect sizes (e.g. Cohen's d, Pearson's r), indicating how they were calculated

Our web collection on statistics for biologists contains articles on many of the points above.

Software and code

Policy information about availability of computer code

Data collection	LSR Fortressa (BD Bioscience), FACSDiva (version 6.1.3, BD Biosciences), Leica DM6000B
Data analysis	DESeq2 package from Bioconductor 3.12<br>Flow cytometry: FlowJo v10<br>Statistical analysis: GraphPad Prism v7 & v8

For manuscripts utilizing custom algorithms or software that are central to the research but not yet described in published literature, software must be made available to editors and reviewers. We strongly encourage code deposition in a community repository (e.g. GitHub). See the Nature Research guidelines for submitting code & software for further information.

Data

Policy information about availability of data

All manuscripts must include a data availability statement. This statement should provide the following information, where applicable:

- Accession codes, unique identifiers, or web links for publicly available datasets
- A list of figures that have associated raw data
- A description of any restrictions on data availability

The data that support the findings of this study are available from the corresponding author upon reasonable request.

Field-specific reporting

Life sciences study design

All studies must disclose on these points even when the disclosure is negative.

Sample size	No statistical method was used to predetermine experimental sample size. Sample size was determined based on our previous studies (Lee et al, 2014; Georgiev et al, 2016) and by availability of experimental animals.
Data exclusions	No data were excluded.
Replication	All attempts at replication were successful. Each data point represents one animal.
Randomization	No randomization was performed. All experimental groups were based on genotype of mice
Blinding	Experiments were not performed blinded since measurements did not involve immediate investigator interpretation.

Reporting for specific materials, systems and methods

We require information from authors about some types of materials, experimental systems and methods used in many studies. Here, indicate whether each material, system or method listed is relevant to your study. If you are not sure if a list item applies to your research, read the appropriate section before selecting a response.

Materials & experimental systems

n/a	Involved in the study	
☐	☒	Antibodies
☐	☒	Eukaryotic cell lines
☒	☐	Palaeontology and archaeology
☐	☒	Animals and other organisms
☒	☐	Human research participants
☒	☐	Clinical data
☒	☐	Dual use research of concern

Methods

n/a	Involved in the study	
☒	☐	ChIP-seq
☐	☒	Flow cytometry
☒	☐	MRI-based neuroimaging

Antibodies

Antibodies used

Antibodies used for flow cytometry analysis:
Anti-mouse CD4 BUV395 (GK1.5) BD Bioscience Cat# 563790
Anti-mouse CD4 BUV737 (GK1.5) BD Bioscience Cat# 564298
Anti-mouse CD8a BUV395 (53-6.7) BD Bioscience Cat# 563786
Anti-mouse CD8a BUV737 (53-6.7) BD Bioscience Cat# 564297
Anti-human HLA-A,B,C PE (W6/32) BioLegend Cat# 311405
Anti-mouse I-A/I-E BV510 (M5/114.15.2) BioLegend Cat# 107635
Anti-mouse H-2Ld/H-2Db PE (28-14-8) BioLegend Cat# 114507
Anti-mouse H-2Kb PE (AF6-88.5) BioLegend Cat# 116508
Anti-mouse Qa-1(b) PE (6A8.6F10.1A6) BD Bioscience Cat# 566640
Anti-mouse Qa-2 FITC (695H1-9-9) BioLegend Cat# 121709
Anti-mouse CD1d PE (1B1) BD Bioscience Cat# 553846
Anti-human/mouse/rat MR1 APC (26.5) BioLegend Cat# 361108
Anti-mouse TCRb BV421 (H57-597) BioLegend Cat# 109230
Anti-mouse/rat Foxp3 PE (FJK-16s) eBioscience Cat# 12-5773-82
Anti-mouse TCR γ/δ PE-Cy7 (GL3) BioLegend Cat# 118124
Anti-mouse PLZF AF647 (R17-809) BD Bioscience Cat# 563490
Anti-mouse PLZF AF488 (Mags.21F7) eBioscience Cat# 53-9320-82
Anti-mouse CD45.1 BV510 (A20) BioLegend Cat# 110741
Anti-mouse CD45.2 BUV737 (104) BD Bioscience Cat# 564880
Anti-mouse RORγt BV786 (Q31-378) BD Bioscience Cat# 564723
Anti-mouse/rat CD44 BV510 (IM7) BD Bioscience Cat# 563114
Anti-mouse NK1.1 FITC (PK136) BioLegend Cat# 108706
Anti-mouse CD138 PE (281-2) BioLegend Cat# 142504
Anti-mouse CD122 PE (TM-β1) BioLegend Cat# 123210
Anti-mouse CXCR3 FITC (CXCR3-173) BioLegend Cat# 126536
Anti-mouse CD69 PE (H1.2F3) eBioscience Cat# 12-0691-83
Anti-mouse CD279 (PD-1) PE (J43) eBioscience Cat# 12-9985-82
Anti-mouse CD196 (CCR6) PE (29-2L17) BioLegend Cat# 129804
Anti-mouse CD25 PE (PC61) BioLegend Cat# 102008
Anti-mouse IFN gamma PE (XMG1.2) eBioscience Cat# 12-7311-82
Anti-mouse IL-4 APC (11B11) BD Bioscience Cat# 554436
Anti-mouse CD19 PerCP-Cy5.5 (eBio1D3) eBioscience Cat# 45-0193-82

	Anti-mouse IL-17A PE (TC11-18H10.1) BioLegend Cat# 506904<br>Anti-mouse Eomes AF488 (Dan11mag) eBioscience Cat# 53-4875-82<br>Anti-mouse V beta 2 TCR FITC (B20.6) BD Bioscience Cat# 553280<br>Anti-mouse V beta 3 TCR FITC (KJ25) BD Bioscience Cat# 553208<br>Anti-mouse V beta 4 TCR FITC (KT4) BD Bioscience Cat# 553365<br>Anti-mouse V beta 5.1/5.2 TCR FITC (MR9-4) BD Bioscience Cat# 553189<br>Anti-mouse V beta 6 TCR FITC (RR4-7) BD Bioscience Cat# 553193<br>Anti-mouse V beta 7 TCR FITC (TR310) BD Bioscience Cat# 553215<br>Anti-mouse V beta 8.3 TCR FITC (1B3.3) BD Bioscience Cat# 553663<br>Anti-mouse V beta 10b TCR FITC (B21.5) BD Bioscience Cat# 553284<br>Anti-mouse V beta 11 TCR FITC (RR3-15) BD Bioscience Cat# 553197<br>Anti-mouse V beta 12 TCR FITC (MR11-1) BD Bioscience Cat# 553300<br>Anti-mouse V beta 13 TCR FITC (MR12-3) BD Bioscience Cat# 553204<br>Anti-mouse V beta 14 TCR FITC (14-2) BD Bioscience Cat# 553258<br>Antibodies used for Immunofluorescence staining:<br>Anti-mouse b5t (polyclonal) MBL International Cat# PD021B<br>Anti-mouse CD16/CD32 (2.4G2) Tonbo Biosciences Cat# 70-0161-U500<br>Goat-anti-Rabbit-AF555 (polyclonal) Thermo Fisher Scientific Cat# A-21428
Validation	All antibodies were validated by their manufacturers for flow cytometry or immunofluorescence microscopy. Validation data for all antibodies are available on the manufacturers website.

Eukaryotic cell lines

Policy information about cell lines

Cell line source(s)	HEK 293T cell line was purchased from Clontech Cat# 632180
Authentication	The HEK 293T cell line was not authenticated.
Mycoplasma contamination	The HEK 293T cell line was not tested for mycoplasma contamination.
Commonly misidentified lines (See ICLAC register)	No commonly misidentified lines were used.

Animals and other organisms

Policy information about studies involving animals; ARRIVE guidelines recommended for reporting animal research

Laboratory animals	C57BL/6NCrl (Strain Code 556) and B6.SJL-PtprcaPepcb/BoyCrCrl (Strain Code 564) mice were obtained from Charles River (via the National Cancer Institute). BALB/cByJ (Stock No: 001026), B6.129S6-Sh2d1atm1Pls/J (Stock No: 025754), C57BL/6-Tg(Lck-CIITA) 16Spark/J (Stock No: 030938) and B6.129S6-Del(3Cd1d2-Cd1d1)1Sbp/J (Stock No: 008881) mice were purchased from Jackson Laboratories. CD8.4 transgenic mice were kindly provided by Dr. Alfred Singer (NCI/CCR, Bethesda). The Nlrc5-stop-flox transgenic mice were described in this paper. All animals used in this study were 6–10 weeks old at the time of analysis. Both male and female mice were used. All animals were maintained under specific pathogen-free conditions at the University of Minnesota.
Wild animals	No wild animals were used.
Field-collected samples	No field-collected samples were used.
Ethics oversight	All experimental procedures were approved by the institutional animal care and use committee at the University of Minnesota (IACUC 1706-34889A and 1709-35136A).

Note that full information on the approval of the study protocol must also be provided in the manuscript.

Flow Cytometry

Plots

Confirm that:

- [x] The axis labels state the marker and fluorochrome used (e.g. CD4-FITC).
- [x] The axis scales are clearly visible. Include numbers along axes only for bottom left plot of group (a 'group' is an analysis of identical markers).
- [x] All plots are contour plots with outliers or pseudocolor plots.
- [x] A numerical value for number of cells or percentage (with statistics) is provided.

Methodology

Sample preparation	Thymi, spleens and livers were harvested and single-cell suspensions were prepared on ice in FACS buffer (PBS/3% FCS) by mechanical dissociation and filtering through 70 um mesh filters. Liver lymphocytes were isolated with Percoll gradient.
Instrument	LSR Fortessa (BD Biosciences)

Software	FacsDIVA (BD Biosciences), FlowJo v10
Cell population abundance	N/A
Gating strategy	All analzyed cell fractions were gated on FSC-A/SSC-A (lymphocytes gate) and then on FSC-H/SSC-W to gate on singlet cells. Next dead cells were excluded by using viability dye.

[x] Tick this box to confirm that a figure exemplifying the gating strategy is provided in the Supplementary Information.
